# How a population-based cohort of men estimate lifetime risk of prostate cancer in a survey before entering a prostate cancer screening trial in Sweden?

**DOI:** 10.1136/bmjopen-2023-083562

**Published:** 2024-08-17

**Authors:** Emmeli Palmstedt, Marianne Månsson, Karin Stinesen Kollberg, Sigrid Carlsson, Mikael Hellström, Jonas Wallström, Jonas Hugosson, Rebecka Arnsrud Godtman

**Affiliations:** 1 Department of Urology, University of Gothenburg Institute of Clinical Sciences, Goteborg, Sweden; 2 Social Work, University of Gothenburg Faculty of Social Science, Gothenburg, Sweden; 3 Department of Surgery and Epidemiology and Biostatistics, Memorial Sloan-Kettering Cancer Center, New York City, New York, USA; 4 Translational Medicine, Division of Urological Cancers, Lund University Medical Faculty, Lund, Sweden; 5 Department of Radiology, Sahlgrenska University Hospital, Gothenburg, Sweden; 6 Department of Radiology, University of Gothenburg Institute of Clinical Sciences, Goteborg, Sweden; 7 Department of Urology, Sahlgrenska University Hospital, Gothenburg, Sweden

**Keywords:** prostate, urological tumours, surveys and questionnaires

## Abstract

**Objectives:**

Investigating men’s perceived lifetime risk of prostate cancer.

**Design:**

Survey-based study to men invited for prostate-specific antigen (PSA) screening in the GÖTEBORG-2 trial between September 2015 and June 2020.

**Setting:**

38 775 men in the Gothenburg area, Sweden, were invited for PSA-testing and participated in a survey.

**Participants:**

17 980 men participated in PSA-testing, of whom 13 189 completed the survey. In addition, 1264 men answered the survey only.

**Interventions:**

Before having the PSA-test, men answered an electronic survey and estimated their lifetime risk of receiving a prostate cancer diagnosis on a visual analogue scale from 0% to 100%.

**Main outcome measures:**

The primary outcome was the median lifetime risk estimation, which was compared with Wilcoxon test to an anticipated lifetime risk of 20% (based on GÖTEBORG-1 trial). The secondary outcome was to determine factors associated with risk estimation in a multivariable linear regression model: previous prostate examination, family history, physical exercise, healthy diet, comorbidity, alcohol consumption, smoking, education level, marital status, urinary symptoms and erectile dysfunction.

**Results:**

Among PSA-tested men, the median estimated lifetime risk of prostate cancer was 30% (IQR 19% to 50%), corresponding to a 10 percentage-points higher estimation compared with the anticipated risk (p<0.001). Family history of prostate cancer, moderate to severe urinary symptoms and mild to moderate erectile dysfunction were associated with >5 percentage-points higher risk estimation. Similar results were obtained for non-PSA-tested men.

**Conclusions:**

Most men overestimated their prostate cancer risk which underscores the importance of providing them accurate information about prostate cancer.

**Trial registration number:**

ISRCTN94604465.

**STRENGTHS AND LIMITATIONS OF THIS STUDY**

- A large population.
- The response rate is high (73%).
- Risk of selection bias since men in this study have chosen to participate in a screening study for prostate cancer.

## Introduction

 Prostate cancer screening with prostate-specific antigen (PSA) continues to be a highly debated topic around the world. The European Randomised Study of Screening for Prostate Cancer (ERSPC) and the GÖTEBORG-1 (G1) trials demonstrated a 20%–30% reduction in prostate cancer-specific mortality at 16–22 years of follow-up.^1^,^3^ The main harms of screening are overdiagnosis and overtreatment with side-effects that may significantly impact men’s quality of life.^24^,^6^ Given the uncertain balance between benefits and harms, most organisations and guidelines recommend that asymptomatic men participate in shared decision making about PSA-testing.^7 8^

Being provided adequate information is important for men to make this informed decision. A key component is having a realistic assessment of the lifetime risk of being diagnosed with prostate cancer. Yet, research is lacking regarding how men perceive their risk of prostate cancer. Available studies report contrasting findings, some studies report that men estimate their risk of prostate cancer to be low,^9 10^ whereas others have found that men overestimate their risk.^10^,^12^

Limited information available on how men participating in a screening study estimate their lifetime risk of receiving a prostate cancer diagnosis. The objective of this study was therefore to investigate how men in the GÖTEBORG-2 trial (G2), a population-based prostate cancer screening trial, estimate their lifetime risk of prostate cancer and whether there are specific factors associated with risk estimation. We hypothesise that men overestimate their lifetime risk of being diagnosed with prostate cancer compared with the real average lifetime risk in a screening population.

## Methods

The GÖTEBORG-2 trial (G2 trial) was approved by the local Ethics Review Committee at the University of Gothenburg in 2015. A detailed description of the G2 trial has been described previously.^13 14^ In brief, the study includes approximately 60 000 men living in Gothenburg and surrounding counties in Sweden. Men aged 50–60 years were randomised into two groups, one invited to a PSA+MRI-based screening and one constituting a control group that was neither invited to the screening nor to the survey (2:1 ratio). Invitation for screening took place between September 2015 and June 2020.

A letter of invitation was sent to all men in the screening group together with a brochure containing information about the G2 trial, risks and benefits and general information about prostate cancer including mentioning that ‘prostate cancer is the most common cancer among Swedish men and also the most common cancer-related cause of death’, but it did not contain any numerical risk estimates. The invitation letter also encouraged men to complete a survey prior to having their initial PSA-test. In the invitation, it was clarified that participation in the screening trial and answering the survey was completely voluntary and that the participant could withdraw or cancel their participation in the study at any time (opt-out model).

By using a unique code provided in the invitation documents, the participant could log in and answer the electronic survey online. A paper version of the survey was also available on request. Men were instructed to complete the survey before having the PSA-test and the online survey was closed when a PSA-result was entered into the database, or after 3 months.

**Figure 1:**
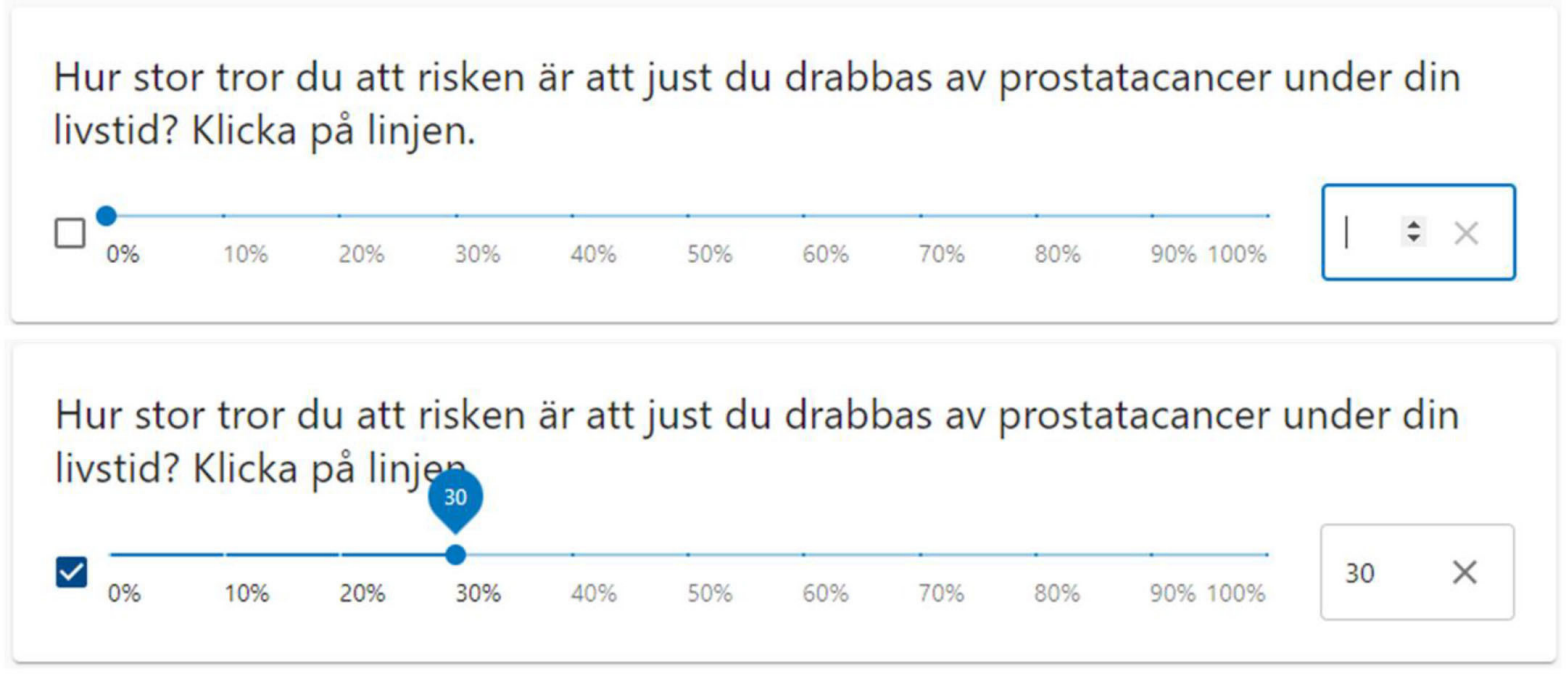
The online version of the perceived risk question. The upper figure demonstrates an unanswered question and the lower being answered to a risk of 30%. A translation of the question: ‘During your lifetime, how high do you estimate that your risk of being diagnosed with prostate cancer is? Mark on the line’.

The survey started with questions about prior PSA testing, prior prostate examinations and family history of prostate cancer (diagnosis in any family member at any age, not only first-degree relatives) (online supplemental document 1). After that, we asked men to estimate their lifetime risk of prostate cancer: ‘During your lifetime, how high do you estimate that your risk of being diagnosed with prostate cancer is?’ Men were asked to estimate their lifetime risk by putting a mark on a visual analogue scale from 0% to 100%, where a number also was displayed while marking (figure 1). Thereafter, followed demographic questions about physical and mental health and background information such as marital status, education, ethnicity, smoking and drinking habits.

**Figure 2:**
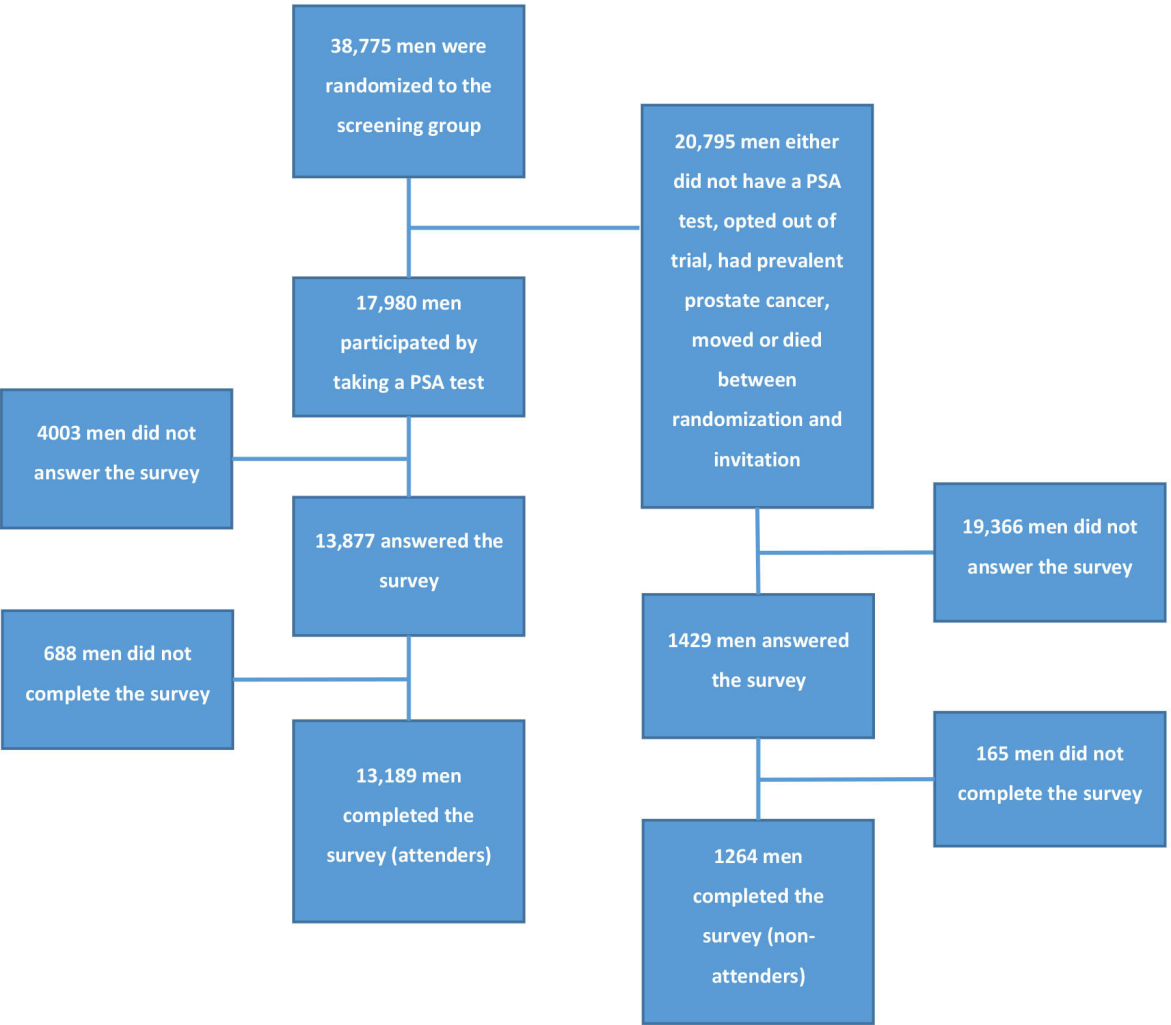
Flowchart of the study population. PSA, prostate-specific antigen.

All men who completed the survey in the first screening round of the G2 trial were included and constituted the study population. Our main focus was on men participating in the screening trial by having a PSA-test (attenders) (figure 2). Men who completed the survey but who did not have a PSA-test (non-attenders) were analysed separately.

### Patient and public involvement

Patients or the public have not been involved in the design, conduct, reporting or dissemination plans of this survey study.

### Statistical methods

The primary outcome was men’s estimated lifetime risk of prostate cancer at the time they responded to the survey, before having a PSA-test. The median of the risk estimates was compared with an anticipated lifetime risk of prostate cancer diagnosis in a screening population, which was set to 20% based on the observed cumulative 24 year incidence of prostate cancer in the screening group (unpublished data) of the GÖTEBORG-1 trial.^15^ Wilcoxon one-sample signed-rank test was used to test whether there was a significant difference between the median of the estimated risk and the anticipated risk. Background characteristics were analysed separately for men attending PSA-testing (attenders) and those not attending PSA-testing (non-attenders), displayed in tables1  2, respectively.

**Table 1:** Background characteristics for the main study population (attenders)

Background characteristics (n=13 189)			
		N	Per cent (%)
Previous prostate examination	Yes	7109	54
	No	5885	45
	Not available	195	1
Family history of prostate cancer	Yes	1817	14
	No	7940	60
	Not available	3432	26
Physical exercise	Several times a week	5788	44
	Once a week	5272	40
	Never	1862	14
	Not available	267	2
Healthy diet	Most commonly	8324	63
	Sometimes	4082	31
	Rarely	719	5
	Not available	64	<1
Comorbidity	Yes	5306	40
	No	7402	56
	Not available	481	4
Current smoker	Yes	1204	9
	No	11 879	90
	Not available	106	1
Alcohol consumption	I never drink alcohol	999	8
	Normal	10 740	81
	Risk consumption	1232	9
	Not available	218	2
Degree of education	Elementary school or equivalent	918	7
	Upper secondary school or equivalent	6291	48
	University or college	5951	45
	Not available	29	<1
Partner	Has partner	10 846	82
	No partner	2310	18
	Not available	33	<1
International prostate symptom score (IPSS)	No or mildly symptomatic	9716	74
	Moderately symptomatic	2346	18
	Severely symptomatic	369	3
	Not available	758	6
IIEF-5 estimation for erectile function	No erectile dysfunction	6816	52
	Mild erectile dysfunction	1606	12
	Mild to moderate erectile dysfunction	491	4
	Moderate erectile dysfunction	174	1
	Severe erectile dysfunction	129	<1
	No sexual activity has occurred	2787	21
	Not available	1186	9

Previous prostate examination was defined as answering ‘yes’ to any of the following: previous PSA-testing, previous prostate biopsy, previous digital rectal examination of the prostate, previous MR imagingI and/or ultrasound of the prostate. Family history of prostate cancer was defined as men answering ‘yes’ to having family history of prostate cancer. Comorbidity was dichotomizsed to ‘yes’ or ‘no’ where men answering ‘yes’ to having/having had one or more of the following diseases: myocardial infarction, angina pectoris, coronary stenosis, heart failure, aortic stenosis, atrial fibrillation, COPD, asthma, diabetes, treatment for high cholesterol, stroke, TIA (transient ischemicischaemic attack), aortic aneurysm, peripheral vascular disease, pulmonary thromboembolism, deep vein thrombosis, and/or blood pressure-lowering medication. Smoking status was set to ‘non-smoker’ or ‘current smoker’ if you had been smoking in the last month. Alcohol consumption was divided into ‘risk consumption’ (defined as drinking 2 or more standard units per day), while less than 2 standard units a day counted as ‘not risk consumption’. LUTS was evaluated by the International Prostate Symptom Score (IPSS) where a score between 0 and 7 was defined as not or mildly symptomatic; 8–19 as moderately symptomatic; 20–35 as severely symptomatic. ED was evaluated by The International Index of Erectile Function (IIEF-5) Questionnaire where scores of 22–25 represented no erectile dysfunction; 17–21 mild erectile dysfunction; 12–16 mild to moderate erectile dysfunction; 8–11 moderate erectile dysfunction; and 5–7 severe erectile dysfunction. Not available (NA) corresponds to the answers ‘Ddo no´’t know/ Ddecline to respond’ or missing answer for a specific variable.

COPDchronic obstructive pulmonary diseaseEDerectile dysfunctionLUTSlower urinary tract symptomsPSAprostate-specific antigen

**Table 2:** Background characteristics for non-attenders (men who answered the survey but did not take a PSA-test)

Background characteristics (n=1264)			
		N	Per cent (%)
Previous prostate examination	Not available	23	2
	Yes	613	48
	No	628	50
Family history of prostate cancer	Yes	152	12
	No	781	62
	Not available	331	26
Physical exercise	Several times a week	485	38
	Once a week	512	41
	Never	239	19
	Not available	28	2
Healthy diet	Most commonly	719	57
	Sometimes	415	33
	Rarely	118	9
	Not available	12	1
Comorbidity	Yes	548	43
	No	653	52
	Not available	63	5
Smoking last month	Yes	224	18
	No	1019	81
	Not available	21	2
Alcohol consumption	I never drink alcohol	141	11
	Normal	939	74
	Risk consumption	152	12
	Not available	32	3
Degree of education	Elementary school or equivalent	107	9
	Upper secondary school or equivalent	627	50
	University or college	523	41
	Not available	7	<1
Partner	Has partner	972	77
	No partner	282	22
	Not available	10	<1
International prostate symptom score (IPSS)	No or mildly symptomatic	933	74
	Moderately symptomatic	189	15
	Severely symptomatic	43	3
	Not available	99	8
IIEF-5 estimation for erectile function	No erectile dysfunction	598	47
	Mild erectile dysfunction	150	12
	Mild to moderate erectile dysfunction	73	6
	Moderate erectile dysfunction	23	2
	Severe erectile dysfunction	9	1
	No sexual activity has occurred	267	21
	Not available	144	11

Previous prostate examination was defined as answering ‘yes’ to any of the following: previous PSA-testing, previous prostate biopsy, previous digital rectal examination of the prostate, previous MR imagingI and/or ultrasound of the prostate. Family history of prostate cancer was defined as men answering ‘yes’ to having family history of prostate cancer. Comorbidity was dichotomizsed to ‘yes’ or ‘no’ where men answering ‘yes’ to having/having had one or more of the following diseases: myocardial infarction, angina pectoris, coronary stenosis, heart failure, aortic stenosis, atrial fibrillation, COPD, asthma, diabetes, treatment for high cholesterol, stroke, TIA (transient ischemicischaemic attack), aortic aneurysm, peripheral vascular disease, pulmonary thromboembolism, deep vein thrombosis, and/or blood pressure-lowering medication. Smoking status was set to ‘non-smoker’ or ‘current smoker’ if you had been smoking in the last month. Alcohol consumption was divided into ‘risk consumption’ (defined as drinking 2two or more standard units per day) while less than 2two standard units a day counted as ‘not risk consumption’. LUTS was evaluated by the International Prostate Symptom Score (IPSS) where a score between 0 and 7 was defined as not or mildly symptomatic; 8–19 as moderately symptomatic; 20–35 as severely symptomatic. ED was evaluated by The International Index of Erectile Function (IIEF-5) questionnaire where scores of 22–25 represented no erectile dysfunction; 17–21 mild erectile dysfunction; 12–16 mild to moderate erectile dysfunction; 8–11 moderate erectile dysfunction; and 5–7 severe erectile dysfunction. Not available (NA) corresponds to the answers ‘Ddo no´’t know/ Ddecline to respond’ or missing answer for a specific variable.

COPDchronic obstructive pulmonary diseaseEDerectile dysfunctionLUTSlower urinary tract symptomsPSAprostate-specific antigen

There were two secondary analyses. In the first, we calculated the proportion of men who estimated their lifetime risk as <15%, between 15% and 25% and >25%. In the second, the association between various factors and men’s perceived lifetime risk was evaluated using linear regression. Potential factors were identified through literature review. After discussion in the research group, the following factors were selected and analysed in a multivariable linear regression model: previous prostate examination, family history of prostate cancer, physical exercise, healthy diet, comorbidity, alcohol consumption, smoking, educational level, marital status, lower urinary tract symptoms (LUTS) and erectile dysfunction (ED). Not available (NA) corresponds to the answers ‘do no’t know/decline to respond’ or missing answer for a specific variable. Definitions for all factors are included in table 1.

A p-value below 5% was considered statistically significant. Note that the estimates and p-values are approximate since not all assumptions of a linear regression are fully satisfied. The assumptions of the regression and a sensitivity analysis based on transformed data, which resulted in very similar results, are discussed in the online supplemental document 2. Statistical analyses were performed using SPSS Statistics, V.27.0.0 (IBM, Armonk, NY, USA) and R Statistical Software (V.4.0.4).

## Results

**Figure 3:**
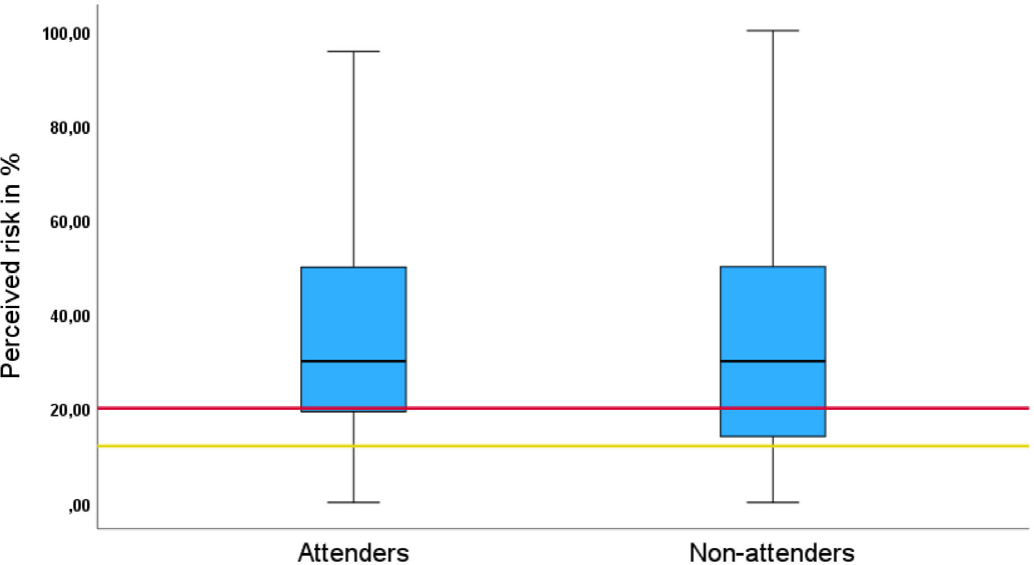
Box plot on men’s lifetime risk estimates of prostate cancer. Attenders, men who responded to the survey and took a PSA-test. Non-attenders, men who responded to the survey but choose not to take a PSA-test. The red line corresponds to the anticipated lifetime risk of 20% and the yellow line corresponds to a lifetime risk of developing symptomatic prostate cancer of 12%. PSA, prostate-specific antigen.

38 775 men were randomised to screening of whom 17 980 chose to participate by having a PSA-test (figure 2). Of these, 73% (13 189/17 980) completed the survey and responded to the question regarding perceived lifetime risk of prostate cancer. The median of the lifetime risk estimations was 30% (IQR 19 to 50) which was significantly higher than the anticipated risk of 20% (p<0.001) (figure 3). Only 19% (2543/13 189) had a risk estimation similar to the anticipated risk, that is, between 15% and 25%, whereas 59% (7743/13 189) estimated their risk to be above 25% and 22% (2903/13 189) of the men estimated their risk to be below 15%. More than 50% (7109/13 189) had previously undergone some form of prostate examination and 14% (1817/13 189) reported a family history of prostate cancer. Additional background characteristics are shown in table 1. Information on background characteristics for subgroups according to risk estimation (below 15%, 15%–25% and above 25%) are shown in online supplemental table S1.

**Figure 4:**
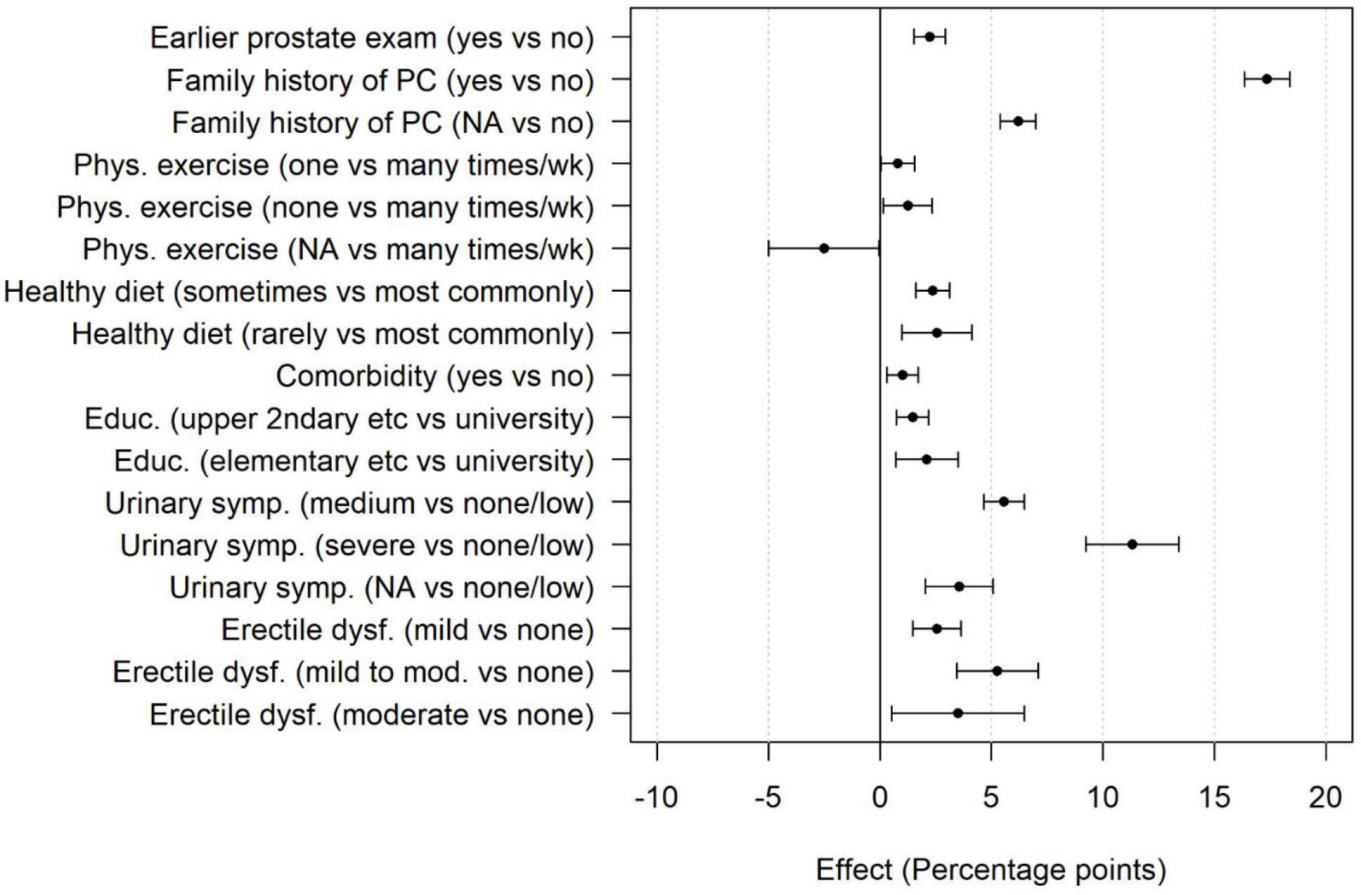
Forrest plot showing factors associated with a significant effect on the risk estimation (in percentage points), displayed with a 95% CI. Not available (NA) corresponds to ‘do no’t know/decline to respond’ or missing answer for a specific variable. PC, prostate cancer.

In the multivariable analysis, factors with a statistically significant association with lifetime risk estimation were as follows: prior prostate examination, family history of prostate cancer, no physical exercise compared with regular physical exercise, rarely or sometimes healthy diet compared with most commonly healthy diet, comorbidity, lower level of education than university or college, moderate to severe LUTS and mild to moderate ED (figure 4). Family history of prostate cancer, moderate to severe LUTS and mild to moderate ED were associated with >5 percentage points higher risk estimation. Factors not statistically significant were smoking, alcohol consumption and intimate partner status. Results are displayed in online supplemental table S2.

Of the 38 775 randomised men, 1264 men answered the survey but chose not to have a PSA-test (non-attenders). These men also estimated their lifetime risk to a median 30% (IQR 14–50), significantly higher than the anticipated risk of 20% (p<0.001) (figure 3). Additional information regarding non-attenders is presented in table 2. The percentage of men estimating their risk to be lower than 15% were 25.8% (326/1264), 14.5% (183/1264) had a risk estimation between 15–25% and 59.7% (755/1264) estimated their risk to be above 25%.

**Figure 5:**
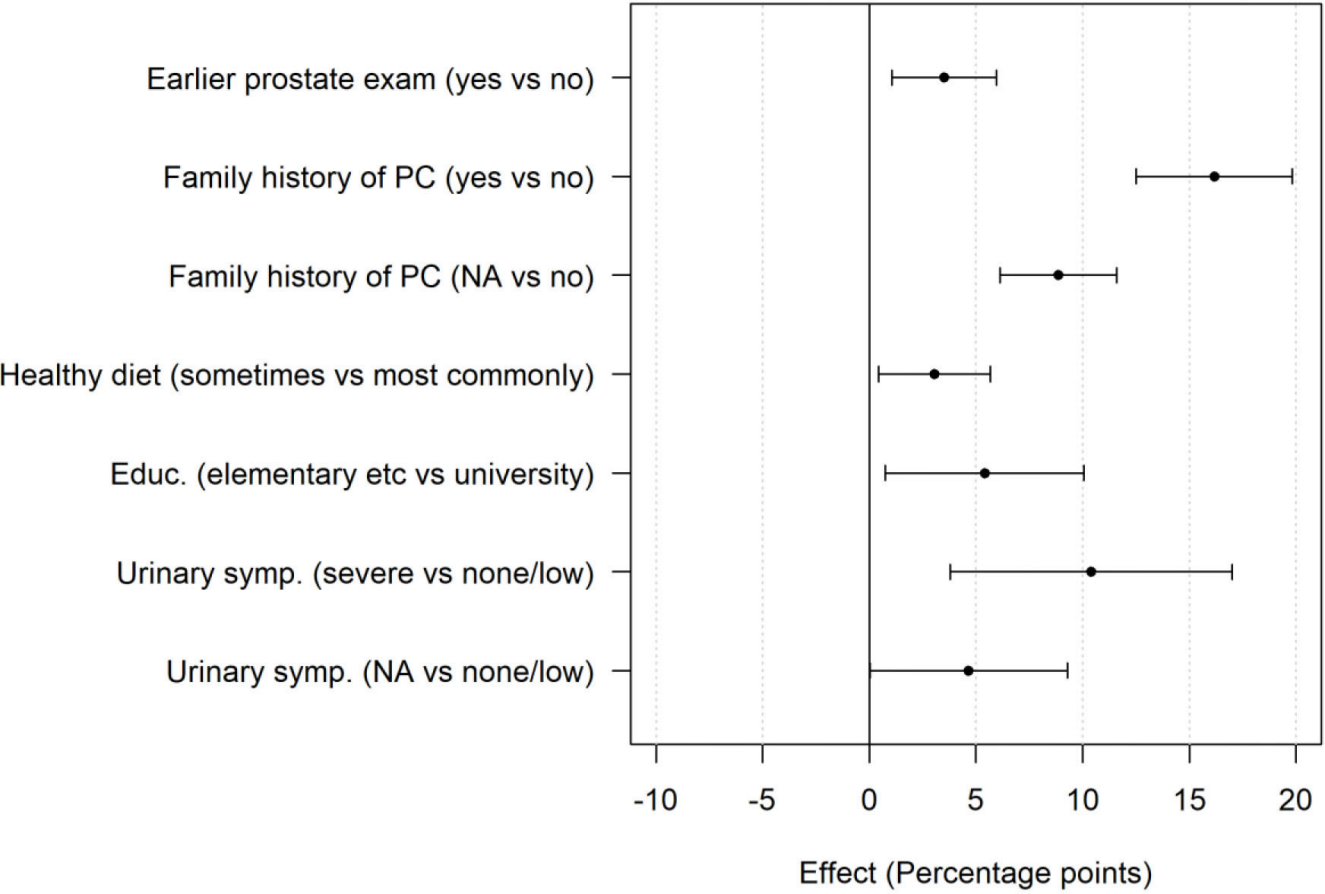
Forrest plot showing factors associated with a significant effect on the risk estimation (in percentage points) for non-attenders (men who answered the survey but did not take a PSA), displayed with a 95% CI. Not available (NA) corresponds to ‘do no’t know/decline to respond’ or missing answer for a specific variable. PC, prostate cancer; PSA, prostate-specific antigen.

In the multivariable analysis for non-attenders, factors with a statistically significant association to lifetime risk estimation were: prior prostate examination, family history of prostate cancer, sometimes healthy diet compared with most commonly healthy diet, lower level of education than university or college, severe LUTS (figure 5). Factors associated with >5 percentage points higher risk estimation were family history of prostate cancer, lower level of education than university or college and severe LUTS. Results are shown in online supplemental table S3.

## Discussion

In this population-based study with more than 13 000 participants, we found that the median of men’s perceived lifetime risk of prostate cancer was approximately 30% which was significantly higher than the average risk in a screening population (20%). Only one in five men estimated their risk between 15% and 25%, and three out of five estimated their risk to be higher than 25%. Hence, on a population level, there is an overestimation, even though for instance men with a family history of prostate cancer are correct in estimating their risk higher than men at average risk. Several factors were significantly associated with perceived risk, including family history of prostate cancer, LUTS and ED which were associated with >5 percentage points higher risk estimation.

To the best of our knowledge, this is the largest survey investigating how men estimate their lifetime risk of prostate cancer. Other strengths include the high response rate (73%) and the fact that men completed the survey at home before the screening procedures. The main limitation is the risk of selection bias since the study population consisted of men who had chosen to participate in a prostate cancer screening trial. These men could potentially perceive their risk differently than the general public since risk perception affects health-seeking behaviour.^16^,^18^ Interestingly, non-attenders’ risk estimation was almost the same as attenders’. The median and upper quartile are identical, while more men among attenders predicted their risk low than among non-attenders as can be seen in figure 3. In the linear regression, the statistically significant factors for non-attenders were also significant for attenders. However, more factors were non-significant for non-attenders. This is partly due to the smaller sample size leading to lower power. For instance, the point estimates for comorbidity were higher for non-attenders than for attenders, although not statistically significant. Altogether, the results show that risk estimation among attenders and non-attenders are similar, indicating that these results can be generalised to men of the same age and background.

Most previous studies investigating perceived risk of prostate cancer have asked men to estimate their risk in categories such as low, average, high^10 19 20^ or as a relative risk compared with the average man^1021^,^24^ rather than a numerical value. Two exceptions were found. The first is a small Swedish study where unaffected men in families with hereditary prostate cancer estimated their own risk to be 50% of receiving a prostate cancer diagnosis and the risk for the general male population to be 33%,^12^ which is much in line with the estimates reported herein. The second is a telephone interview-based, Australian study in which men estimated their own lifetime risk of prostate cancer to be 25% and the risk for an average man to be 32%.^25^

Risk assessment is for many people a difficult concept. A limitation of this study is therefore that the risk assessment was done only by a one-item numerical rating scale from 0% to 100%. In previous studies in breast cancer, this type of measurement often resulted in overestimating the risk, because the anchors were sometimes misleading, and some women perceived their risk of developing breast cancer as 50%, not understanding that this means a one in two chance.^26^ However, we acknowledge that there is no gold standard measurement for perceived risk and the one-item numerical rating scale is a validated instrument with acceptable psychometric properties that outperforms other items and formats and is frequently used.^27 28^

There were other questions regarding how well men understood the concept of lifetime risk. Many men probably interpret lifetime risk of prostate cancer as the lifetime risk of developing symptomatic prostate cancer, not being aware that participating in PSA screening also increases the risk of overdiagnosis of asymptomatic cancer. This speculation is supported by the fact that men who choose not to have a PSA-test estimated their risk almost identical to men who were PSA-tested. The lifetime risk of symptomatic prostate cancer before the PSA-era has been estimated to approximately 11%–13% which means that if men interpreted the question as lifetime risk of symptomatic prostate cancer, the overestimation was even larger (figure 3).^29^,^31^ We based the anticipated risk of prostate cancer on the observed 24 year cumulative incidence of prostate cancer in the screening group of the G1 trial in order to have a risk estimate from a screening setting. Even though this is not a lifetime risk since not all men in the G1 trial have died, we believe that it is a fair estimate since the incidence curve has flattened out due to deaths from competing events (unpublished data).^15^ Figures similar to our estimate have also been published by others.^29 32^

We can only speculate why men in our study overestimated their risk of prostate cancer. Public campaigns aiming at raising awareness about prostate cancer and the invitation letter mentioning ‘prostate cancer is the most common cancer among Swedish men’ may influence men’s perception. Family history of prostate cancer is a well-known risk factor for prostate cancer and in some studies, it has been associated with a two- to threefold higher risk for prostate cancer.^33^,^35^ The higher estimated risk in men reporting a family history of prostate cancer could therefore be an adequate risk estimation for these men. Men with symptoms (LUTS and/or ED) also estimated their risk higher. This finding is not surprising since men often associate urinary symptoms with prostate cancer and is often included in patient information materials.^36^ For example, on the official information website for the Swedish healthcare system*,* frequency, a weak stream and urinary hesitance are listed as typical symptoms of prostate cancer.^37^ Most men with LUTS however, do not suffer from prostate cancer but rather have benign prostate enlargement and the association between LUTS and prostate cancer is weak.^38^,^40^

The inconsistency between the perceived and actual risk of developing prostate cancer is an important finding for several reasons. First, since higher perceived risk is linked to higher worry for cancer,^41^ men’s overestimation could mean that they worry about prostate cancer unnecessarily and improved information could potentially reduce this anxiety. Second, if men do not have an accurate risk estimate, they cannot make a truly informed decision as to whether to be screened for prostate cancer or not. Third, given our results that men associate LUTS with prostate cancer risk, this is a group that particularly could benefit from improved information. Many men may worry about prostate cancer since LUTS are very common.^42^ These men may decide to have a PSA-test based on the assumption that they are at risk for prostate cancer, while those without urinary dysfunction may abstain from testing themselves in a false sense of security, believing that they are not at risk for prostate cancer, potentially leading to a delayed diagnosis.

Our findings show that men overestimate their risk of prostate cancer, and thereby potentially also overestimate the benefit of screening which is in line with other reports showing that both patients and healthcare workers tend to overestimate the benefits from medical interventions.^43 44^ Thus, there is a need to improve information concerning prostate cancer risk, risk of overdiagnosis and outcomes after medical interventions helping men to get a realistic risk perception. Prediction models may help to obtain personalised risk estimates. Such improved information could be communicated using icon arrays and numerical risk estimates, so that men can make a well-informed decision on whether to be screened or not for prostate cancer.^45^ Urinary symptoms is a common reason why men ask for PSA-testing but the link between urinary symptoms and prostate cancer should be removed^36 40^ and the public messaging should instead emphasise that prostate cancer commonly is silent or asymptomatic, particularly in the curable stages of the disease. Men with urinary symptoms should have the same information regarding benefits and harms of screening for prostate cancer.

### Conclusion

We found that men choosing to participate in prostate cancer screening overestimate their risk of prostate cancer, only one in five men had a risk estimate close to the anticipated risk. In order for men to truly make an informed decision on whether to be screened for prostate cancer or not, an adequate risk perception is a key component. Our findings, therefore, highlight the need for improved information concerning prostate cancer risk.

## supplementary material

10.1136/bmjopen-2023-083562 online supplemental file 1

10.1136/bmjopen-2023-083562 online supplemental file 2

10.1136/bmjopen-2023-083562 online supplemental file 3

10.1136/bmjopen-2023-083562 online supplemental file 4

10.1136/bmjopen-2023-083562 online supplemental file 5

## Data Availability

Data are available upon reasonable request.
