# Supplementary material for: How a population-based cohort of men estimate lifetime risk of prostate cancer in a survey before entering a prostate cancer screening trial in Sweden?
Source: BMJ Open. 2024 Aug 17;14(8):e083562. doi: 10.1136/bmjopen-2023-083562 (PMC11331866; doi:10.1136/bmjopen-2023-083562)
Supplement: online supplemental file 1 [file bmjopen-14-8-s001.pdf]

SAHLGRENSKA AKADEMIN

QUESTIONNAIRE 1

GÖTEBORG-2-TRIAL

QUESTIONS ABOUT YOUR HEALTH AND WELL-BEING

Studieid:

Datum: □□□□-□□-□□

År Mån Dag

Vänligen fyll i det datum då du besvarar enkäten

This questionnaire contains general questions about your health and some background questions about you. Please answer the questions by marking the response alternative that best corresponds to you. All your responses will be analyzed anonymously. **Please note that this questionnaire cannot be used to communicate other health information. For that, you need to contact your primary care facility.** ***Thank you for your participation!***

Background questions

Prostate investigation history

1. Have you ever undergone any prior clinical investigation related to your prostate? ◯ Yes ◯ No ◯ **Don't know/Decline to respond**

If yes...

I have taken a PSA test (blood draw)

◯ **Once**
◯ **More than once**
◯ **Never**
◯ **Don't know/Decline to respond**

If once/more than once...

How high was your *most recent* PSA value?

_________ **ng/ml**

◯ **Don't know/Decline to respond**

My physician has examined my prostate (rectal examination using finger) ◯ **Yes** ◯ **No** ◯ **Don't know/Decline to respond**

If yes…

What was the result of the examination? You may check more than one alternative.

◯ **Normal findings** ◯ **Enlarged prostate** ◯ **Inflammation**

◯ **Prostate cancer** ◯ **Don't know** ◯ **Other:** __________________

I have had my prostate examined by ultrasound previously ◯ **Yes** ◯ **No** ◯ **Don't know/Decline to respond**

If yes…

What was the result of the examination? You may check more than one alternative.

◯ **Normal findings** ◯ **Enlarged prostate** ◯ **Inflammation**

◯ **Prostate cancer** ◯ **Don't know** ◯ **Other:** ___________________

I have had a prostate biopsy previously ◯ **Yes** ◯ **No** ◯ **Don't know/Decline to respond**

If yes…

What was the result of the examination? You may check more than one alternative.

◯ **Normal findings** ◯ **Enlarged prostate** ◯ **Inflammation**

◯ **Prostate cancer** ◯ **Don't know** ◯ **Other:** ___________________

I have had my prostate examined by MRI (Magnetic Resonance Imaging) previously ◯ **Yes** ◯ **No** ◯ **Don't know/Decline to respond**

If yes…

What was the result of the examination? You may check more than one alternative.

◯ **Normal findings** ◯ **Enlarged prostate** ◯ **Inflammation**

◯ **Prostate cancer** ◯ **Don't know** ◯ **Other:** ___________________

Other previous prostate examination, please state which type:

____

If yes…

What was the result of the examination? You may check more than one alternative.

◯ **Normal findings** ◯ **Enlarged prostate** ◯ **Inflammation**

◯ **Prostate cancer** ◯ **Don't know** ◯ **Other:** ___________________

Heredity

2. Do you have any heredity for prostate cancer? You may check more than one alternative.

◯ **Yes, my father was diagnosed with prostate cancer.**

If yes...

◯ before age 70
◯ after age **70**

◯ **Yes, a brother was diagnosed with prostate cancer.**

If yes...

◯ Before age 70
◯ **after age 70**

◯ **Yes, <u>two or more brothers</u> were diagnosed with prostate cancer.**

If yes...

◯ before age 70
◯ after age 70

◯ **Other family member, namely:**

____

◯ **No**

◯ **Don't know/Decline to respond**

Risk of being diagnosed

3. During your lifetime, how high do you estimate that your risk of being diagnosed with prostate cancer is? Mark with an X on the line:

0% 10% 20% 30% 40% 50% 60% 70% 80% 90% 100%

◯ **Don't know/Decline to respond**

Health activities

4. How often do you engage in the following health activities?

**Health check-ups**	◯ **Yearly**	◯ **Less than once per year**	◯ **Never**	◯ **Don't know/Decline to respond**
**Dentist/dental hygienist**	◯ **Yearly**	◯ **Less than once per year**	◯ **Never**	◯ **Don't know/Decline to respond**
**I practice some kind of physical excercise**	◯ **Several times per week**	◯ **About once per week**	◯ **Never**	◯ **Don't know/Decline to respond**
**I consider my diet as healthy**	◯ **Most commonly**	◯ **Sometimes**	◯ **Rarely**	◯ **Don't know/Decline to respond**

Comorbidities

Källa: Modifierad MALE model enligt A. Vickers et al.

Heart disease

5.	Were you ever told that there is a problem with your heart or were you ever treated for heart disease, chest pain, or angina?	◯ **Yes**	◯ **No**	◯ **Don't know/Decline to respond**

If yes...

Angina
Have you ever experienced unexplainable chest pain or have you received the diagnosis angina, angina pectoris, or coronary artery disease? ◯ **Yes** ◯ **No** ◯ **Don't know/Decline to respond**

Coronary stenosis
Have you undergone bypass surgery or balloon dilation due to narrowing of the coronary arteries of the heart? ◯ **Yes** ◯ **No** ◯ **Don't know/Decline to respond**

Heart failure
Have you received the diagnosis heart failure or pulmonary edema or have you ever been prescribed diuretic medication for your heart? ◯ **Yes** ◯ **No** ◯ **Don't know/Decline to respond**

Myocardial infarction
Have you had a myocardial infarction or heart attack? ◯ **Yes** ◯ **No** ◯ **Don't know/Decline to respond**

Aortic stenosis
Have you received the diagnosis aortic stenosis or narrowing of the aorta in the heart? ◯ **Yes** ◯ **No** ◯ **Don't know/Decline to respond**

Atrial fibrillation
Have you received the diagnosis atrial fibrillation or irregular heart rythm? ◯ **Yes** ◯ **No** ◯ **Don't know/Decline to respond**

Lung disease

6.1 Have you received the diagnosis asthma? ◯ **Yes** ◯ **No** ◯ **Don't know/Decline to respond**

If yes… How severe is your asthma?

◯ **Mild = No impact on my daily activities** ◯ **Moderate = Some impact on my daily activities** ◯ **Severe = Severe impact on my daily activities** ◯ **Don't know/Decline to respond**

6.2 Have you received the diagnosis COPD or emphysema? ◯ **Yes** ◯ **No** ◯ **Don't know/Decline to respond**

Vascular disease

7. **Aortic aneurysm**
Have you received the diagnosis aortic aneurysm or were you ever told that you have a dilation of the corporal artery in your bowel? ◯ **Yes** ◯ **No** ◯ **Don't know/Decline to respond**

8. **Periferal vascular disease**
Have you ever felt pain in your leg(s) due to poor circulation or have you received the diagnosis peripheral vascular disease? ◯ **Yes** ◯ **No** ◯ **Don't know/Decline to respond**

9. **Deep vein thrombosis**

Have you had deep vein thrombosis (blood clot) or have you been treated with blood thinning medication för blood clots in arms or legs?

◯ **Yes** ◯ **No** ◯ **Don't know/Decline to respond**

10. **Pulmonary embolism**

Have you had pulmonary embolism (blood clot) in your lungs?

◯ **Yes** ◯ **No** ◯ **Don't know/Decline to respond**

11. **TIA**

Have you had a TIA-attack (transient ischemic attack) or been treated with blood thinning medication for this?

◯ **Yes** ◯ **No** ◯ **Don't know/Decline to respond**

12. **Stroke**

Have you had a stroke?

◯ **Yes** ◯ **No** ◯ **Don't know/Decline to respond a**

If yes...

Do you know if the stroke was a bleed or an infarct (clot, thrombosis, or embolism) in the brain?

◯ **Cerebral haemorrhage (bleed)** ◯ **Cerebral infacrction** ◯ **Don't know**

Diabetes

13. Have you received the diagnosis diabetes?

◯ **Yes** ◯ **No** ◯ **Don't know/Decline to respond**

If yes...

When did you receive the diagnosis of diabetes?

◯ **0-5 years ago** ◯ **6-10 years ago** ◯ **11-20 years ago** ◯ **> 20 years ago** ◯ **Don't know/Decline to respond**

What treatment do you use for your diabetes? You may check more than one alternative.

◯ **Diet** ◯ **Tablets** ◯ **Insulin** ◯ **Don't know/Decline to respond**

Cholesterol

14. Do you take medication to lower your cholesterol?

◯ **Yes** ◯ **No** ◯ **Don't know/Decline to respond**

Do you know your approximate total cholesterol value? If you take medication to lower your cholesterol, what is your total cholesterol value when taking these tablets?

◯ Very high **(>7.0 mmol/l)** ◯ **High (5.2-7.0 mmol/l)** ◯ **Normal (< 5.2 mmol/l)** ◯ **Don't know/Decline to respond**

Do you know the approximate value of your HDL (the "good" cholesterol)?

◯ **Very low (<0.5 mmol/l)** ◯ **Low (0.5-1.6 mmol/l)** ◯ **Normal (>1.6 mmol/l)** ◯ **Don't know/Decline to respond**

Blood pressure

15. Do you take any medication to lower your blood pressure? ◯ **Yes** ◯ **No** ◯ **Don't know/Decline to respond**

Blood pressure is described in the form of "systolic/diastolic", for example "130/80". If you know your approximate blood pressure, what is your systolic blood pressure? If you take medication to lower your blood pressure, please state your blood pressure when you take these medications.

◯ **High (>= 160 mmHg)** ◯ **Elevated (140-159 mmHg)** ◯ **Normal/low (<140 mmHg)** ◯ **Don't know/Decline to respond**

What is your diastolic pressure?

◯ **High (>= 100 mmHg)** ◯ **Elevated (90-100 mmHg)** ◯ **Normal/low (<90 mmHg)** ◯ **Don't know/Decline to respond**

Additional background questions

Smoking history

16. In total, have you smoked more than 100 cigarettes in your life? ◯ **Yes** ◯ **No** ◯ **Don't know/Decline to respond**

If yes...

Have smoked *within the past month*? ◯ **Yes** ◯ **No** ◯ **Don't know/Decline to respond**

If yes...

At what age did you *start* to smoke? **Age: __________years old** ◯ **Don't know/Decline to respond**

On average, how many cigarettes do you smoke per day?

◯ **Less than 6** ◯ **6-10** ◯ **11-15** ◯ **16-20** ◯ **21-25** ◯ **26-30** ◯ **31-40**

◯ **41-50** ◯ **51-60** ◯ **61-70** ◯ **71-80** ◯ **More than 80** ◯ **Don't know/Decline to respond**

If no...

At what age did you *start* to smoke? **Age: __________years old** ◯ **Don't know/Decline to respond**

On average, how many cigarettes did you smoke per day?

◯ **Less than 6** ◯ **6-10** ◯ **11-15** ◯ **16-20** ◯ **21-25** ◯ **26-30** ◯ **31-40**

◯ 41-50 ◯ 51-60 ◯ 61-70 ◯ 71-80 ◯ More than 80 ◯ Don't know/Decline to respond

At what age did you *quit* smoking? **Age: __________years old** ◯ **Don't know/Decline to respond**

Alcohol consumption

17. *During one week,* how many "glasses" of alcohol or "beer" do you typically drink?

One "glass" is referred to as one glass of white or red wine (10-15 cl); one glass of fortified wine (5-8 cl); one shot (4 cl); or the equivalent amount of hard liquor.

One "beer" is referred to as one bottle of beer (50 cl folk beer or 33 cl strong beer).

◯ I never drink alcohol. ◯ Fewer than **1-2 "glasses" / "beer" per week** ◯ **1-2 "glasses" / "beer"per week**

◯ **Ca 1 "glass" / "beer"per day** ◯ **Ca 2-3 "glasses" / "beer"per day** ◯ **4 or more "glasses" / "beer"per day** ◯ **Don't know/Decline to respond**

Education

18. What is your educational level? Choose the highest level of education attended.

◯ Secondary school or equivalent ◯ High school or equivalent ◯ University or college ◯ **Don't know/Decline to respond**

Employment status

19. Current source of income: ◯ **Full-time work** ◯ **Part-time work, ________% out of full-time work**

◯ **Student or job seeker** ◯ **Early retirement/disability pension** ◯ **Other** ◯ **Don't know/Decline to respond**

Income

20. What is your monthly income in kronor (*before* income tax is deducted)?

◯ **< 20 000 kronor** ◯ **20 000 – 40 000 kronor** ◯ **> 40 000 kronor** ◯ **Don't know/Decline to respond**

Living alone

21. ◯ **Yes** ◯ **No** ◯ **Don't know/Decline to respond**

Marital status

22. Are you currently: ◯ **Not married** ◯ **Married / cohabitating / in a relationship but not living together**

◯ **Divorced** ◯ **Widower** ◯ **Don't know/Decline to respond**

Country of birth

23. Were you born in Sweden? ◯ **Yes** ◯ **No** ◯ **Don't know/Decline to respond**

If no... Country in which you were born: ______________________________

How old you were when you moved to Sweden: ___________ **years old**

**Language**				
24.	Is Swedish your first language?	◯ **Yes**	◯ **No**	◯ **Don't know/Decline to respond**
	**If no...** What is your first language? _____________________________			
25.	Did you need help to fill out this questionnaire?	◯ **Yes**	◯ **No**	◯ **Don't know/Decline to respond**

Urinary symptoms

Källa: International Prostate Symptom Score (IPSS)

Questions about your urinary function (how easy it is for you to pee). Please choose the alternative that best corresponds to your experience _during the past month_.

26. Over the past month, how often have you had a sensation of not emptying your bladder completely after you finish urinating?

Not at all	Less than 1 time in 5	Less than half the time	About half the time	More than half the time	Almost always	Don't know/Decline to respond
◯0	◯1	◯2	◯3	◯4	◯5	◯

27. Over the past month, how often have you had to urinate again less than two hours after you finished urinating?

Not at all	Less than 1 time in 5	Less than half the time	About half the time	More than half the time	Almost always	Don't know/Decline to respond
◯0	◯1	◯2	◯3	◯4	◯5	◯

28. Over the past month, how often have you found you stopped and started again several times when you urinated?

Not at all	Less than 1 time in 5	Less than half the time	About half the time	More than half the time	Almost always	Don't know/Decline to respond
◯0	◯1	◯2	◯3	◯4	◯5	◯

29. Over the past month, how difficult have you found it to postpone urination?

Not at all	Less than 1 time in 5	Less than half the time	About half the time	More than half the time	Almost always	Don't know/Decline to respond

◯0	◯1	◯2	◯3	◯4	◯5	◯

30. Over the past month, how often have you had a weak urinary stream?

Not at all	Less than 1 time in 5	Less than half the time	About half the time	More than half the time	Almost always	Don't know/Decline to respond
◯0	◯1	◯2	◯3	◯4	◯5	◯

31. Over the past month, how often have you had to push or strain to begin urination?

Not at all	Less than 1 time in 5	Less than half the time	About half the time	More than half the time	Almost always	Don't know/Decline to respond
◯0	◯1	◯2	◯3	◯4	◯5	◯

32. Over the past month, how many times did you most typically get up to urinate froom the time you went to bed until the time you got up in the morning?

None	1 time per night	2 times per night	3 times per night	4 times per night	5 times or more per night	Don't know/Decline to respond
◯0	◯1	◯2	◯3	◯4	◯5	◯

33. If you were to spend the rest of your life with your urinary condition the way it is now, how would you feel about that?

Delighted	Pleased	Mostly satisfied	Mixed: Equally satisfied/ dissatisfied	Mostly dissatisfied	Unhappy	Terrible	Don't know/Decline to respond
◯0	◯1	◯2	◯3	◯4	◯5	◯6	◯

33b. Do you *currently* take any kind of medication to facilitate urination? ◯ **Yes** ◯ **No** ◯ **Don't know/Decline to respond**

If yes, please state which medication(s). You may check more than one alternative:

◯ **Finasterid** ◯ **Proscar** ◯ **Dutasterid** ◯ **Avodart**

◯ **Other, state which**____

____

33c. Do you *currently* take any kind of health supplement to facilitate urination? ◯ **Yes** ◯ **No** ◯ **Don't know/Decline to respond**

If yes, please state which health supplement(s):

____

____

Sexual lust

The prostate is a gland which plays a central role to a man's sexual health. Therefore, we wish to ask a few questions about it. We wish to remind you that your responses are anonymous and will be treated with confidentiality.

34. How strong do you experience your current sexual lust to be?

◯ **Very strong** ◯ **Strong** ◯ **Neither strong nor weak** ◯ **Weak** ◯ **Very weak** ◯ **Don't know/Decline to respond**

35. In recent years, have you experienced a decrease in your sexual lust?

◯ Not decreased at all ◯ Somewhat decreased ◯ Much decreased ◯ **Don't know/Decline to respond**

36. How important is your sexuality for you today?

◯ Very important ◯ Quite important ◯ Not important at all ◯ **Don't know/Decline to respond**

Sexual function

These questions are about your sexual function, which includes foreplay, masturbation, intercourse with partner, and other forms of sexual activity *during the past month*.

**Erection aid**			
37. Do you use injections/shots in penis (e.g., Caverject) or use a pin that is inserted through the urethra (e.g.,	◯ **Yes**	◯ **No**	◯ **Don't know/Decline to respond**

Bondil)?

If yes...

In the past month, when you used injections/shots in penis and had an erection with sexual stimulation, how often was your erection stiff enough to penetrate your partner?

No sexual activity	Almost never / Never	A few times (much less than half the time)	Sometimes (about half the time)	Most times (much more than half the time)	Almost always/ Always	Don't know/Decline to respond
◯0	◯1	◯2	◯3	◯4	◯5	◯

38. *In the past month,* how often have you taken a tablet such as Viagra, Cialis or Levitra?	◯ **Never**	◯ **Sometimes**	◯ **Regularly**	◯ **Don't know/Decline to respond**

Kindly, answer the following questions based on your current sexual function WITHOUT any eventual tablets and/or injections/shots if you use any of those.

Källa: International Index of Erectile Function-5 (IIEF-5)

39. In the *past month*, how do you rate your confidence that you could get and keep an erection?

Very low	Low	Moderate	High	Very high	Don't know/Decline to respond
◯1	◯2	◯3	◯4	◯5	◯

40. In the *past month*, when you had erections with sexual stimulation, how often were your erections *hard enough* for penetration?

No sexual activity	Almost never/ Never	A few times (much less than half the time)	Sometimes (about half the time)	Most times (much more than half the time)	Almost always/ Always	Don't know/Decline to respond
◯0	◯1	◯2	◯3	◯4	◯5	◯

41. In the *past month*, during sexual intercourse, *how often* were you able to maintain your erection after you had penetrated (entered) your partner?

No sexual activity	Almost never/ Never	A few times (much less than half the time)	Sometimes (about half the time)	Most times (much more than half the time)	Almost always/ Always	Don't know/Decline to respond
◯0	◯1	◯2	◯3	◯4	◯5	◯

42. In the *past month*, during sexual intercourse, *how difficult* was it to maintain your erection to completion of intercourse?

No sexual activity	Extremely difficult	Very difficult	Difficult	Slightly difficult	Not difficult	Don't know/Decline to respond
◯0	◯1	◯2	◯3	◯4	◯5	◯

43. In the *past month*, when you attempted sexual intercourse, *how often* was it satisfactory for you?

No sexual activity	Almost never/ Never	A few times (much less than half the time)	Sometimes (about half the time)	Most times (much more than half the time)	Almost always/ Always	Don't know/Decline to respond
◯0	◯1	◯2	◯3	◯4	◯5	◯

44. If you were to spend the rest of your life with your sexual function the way it is now, how would you feel about that?

Delighted	Pleased	Mostly satisfied	Mixed: Equally satisfied/ dissatisfied	Mostly dissatisfied	Unhappy	Terrible	Don't know/Decline to respond
◯0	◯1	◯2	◯3	◯4	◯5	◯6	◯

General health and quality of life

45. *In the past month*, how would you rate your general health? Please, mark your response with an X on the line:

Very poor **Very good**

0 1 2 3 4 5 6 7 8 9 10

◯ **Don't know/Decline to respond**

46. *In the past month*, how would you rate your quality of life? Please, mark your response with an X on the line:

Very poor **Very good**

0 1 2 3 4 5 6 7 8 9 10

◯ **Don't know/Decline to respond**

History of sexual activity

47. How old were you when you had sexual intercourse (penetration) for the first time in your life?

Younger than 15 years old	15-18 years old	19-25 years old	Older than 25 years old	I have never had sexual intercourse	Don't know/Decline to respond
○	○	○	○	○	○

48. In your life, how many partners approximately have you had sexual intercourse with?

0	1-2	3-10	11-20	More than 20	Don't know/Decline to respond
○	○	○	○	○	○

Personality

49.	Are you a worrier?	○ Yes	○ No	○ Don't know/Decline to respond
50.	Are your feelings easily hurt?	○ Yes	○ No	○ Don't know/Decline to respond
51.	Do you tend to lose interest in things after a while?	○ Yes	○ No	○ Don't know/Decline to respond
52.	Do you worry too long after an embarrassing experience?	○ Yes	○ No	○ Don't know/Decline to respond
53.	Do you often feel ”fed-up” for no reason?	○ Yes	○ No	○ Don't know/Decline to respond
54	Do you worry that something awful is going to happen?	○ Yes	○ No	○ Don't know/Decline to respond

Subjectiv loneliness

55. I can call on my friends whenever I need them.

Agree completely	Agree to some extent	Disagree to some extent	Disagree completely	Decline to respond
○	○	○	○	○

56. I feel as if nobody really understands me.

Agree completely	Agree to some extent	Disagree to some extent	Disagree completely	Decline to respond
○	○	○	○	○

57. There is no one in my family that I can trust whenever I need help and support.

Agree completely	Agree to some extent	Disagree to some extent	Disagree completely	Decline to respond
◯	◯	◯	◯	◯

58. I feel close to my family.

Agree completely	Agree to some extent	Disagree to some extent	Disagree completely	Decline to respond
◯	◯	◯	◯	◯

59. I have a partner with whom I share my innermost thoughts and feelings.

Agree completely	Agree to some extent	Disagree to some extent	Disagree completely	Don't know/Decline to respond
◯	◯	◯	◯	◯

60. I feel frustrated about not being in an intimate relationship.

Agree completely	Agree to some extent	Disagree to some extent	Disagree completely	Don't know/Decline to respond
◯	◯	◯	◯	◯

Objective loneliness

61. During one week, how many evenings do you typically spend all by yourself?

All evenings	5-6 evenings	3-4 evenings	1-2 evenings	No evening	Don't know/Decline to respond
◯	◯	◯	◯	◯	◯

62. Are you in an intimate relationship?

Yes	No	Don't know/Decline to respond
◯	◯	◯

63. How many people live in your household?

I live alone	I live with at least one person (adult/child)	Decline to respond

64. In the past 12 months, how often have you socialized with friends?

Not once	About twice	About 4 times	About once per month	About once per week	Several times per week	Don't know/Decline to respond
○	○	○	○	○	○	○

HADS *(Hospital Anxiety and Depression Scale)*

Read each statement and check the alternative that best corresponds to how you have been feeling during the past week. Don't think about your response for too long; your spontaneous reaction to each statement is probably more correct than a response you've contemplated for a long time.

To what extent do these statements apply to you?

65. **I feel tense or "wound up"**

Most of the time	A lot of the time	From time to time, occasionally	Not at all	**Don't know/Decline to respond**
○	○	○	○	○

66. **I still enjoy the things I used to enjoy**

Definitely as much	Not quite so much	Only a little	**Hardly at all**	**Don't know/Decline to respond**
○	○	○	○	○

67. **I get a sort of frightened feeling as if something awful is about to happen**

Very definitely and quite badly	Yes, but not too badly	A little, but it doesn't worry me	Not at all	**Don't know/Decline to respond**
○	○	○	○	○

68. **I can laugh and see the funny side of things**

As much as I always could	Not quite so much now	Definitely not so much now	Not at all	**Don't know/Decline to respond**
○	○	○	○	○

69. **Worrying thoughts go through my mind**

A great deal of the time	A lot of the time	From time to time, but not too often	Only occasionally	**Don't know/Decline to respond**
○	○	○	○	○

70. **I feel cheerful**

Not at all	Not often	Sometimes	Most of the time	**Don’t know/Decline to respond**
○	○	○	○	○

71. **I can sit at ease and feel relaxed**

Definitely	Usually	Not Often	Not at all	**Don’t know/Decline to respond**
○	○	○	○	○

72. **I feel as if I am slowed down**

Nearly all the time	Very often	Sometimes	Not at all	**Don’t know/Decline to respond**
○	○	○	○	○

73. **I get a sort of frightened feeling like 'butterflies' in the stomach**

Not at all	Occasionally	Quite Often	Very Often	**Don’t know/Decline to respond**
○	○	○	○	○

74. **I have lost interest in my appearance**

Definitely	I don't take as much care as I should	I may not take quite as much care	I take just as much care as ever	**Don’t know/Decline to respond**
○	○	○	○	○

75. **I feel restless as if I have to be on the move**

Very much indeed	Quite a lot	Not very much	Not at all	**Don’t know/Decline to respond**
○	○	○	○	○

76. **I look forward with enjoyment to things**

As much as I ever did	Rather less than I used to	Definitely less than I used to	Hardly at all	**Don't know/Decline to respond**
◯	◯	◯	◯	◯

77. **I get sudden feelings of panic**

Very often indeed	Quite often	Not very often	Not at all	**Don't know/Decline to respond**
◯	◯	◯	◯	◯

78. **I can enjoy a good book or a radio or TV program**

Often	Sometimes	Not often	**Very seldom**	**Don't know/Decline to respond**
◯	◯	◯	◯	◯

Closing questions

79. Did any of the questions make you feel worried?

◯ **No** ◯ **Yes, a little** ◯ **Yes, a lot** ◯ **Don't know/Decline to respond**

Om ja...

Vilken/vilka frågor och varför:

____

____

____

____

____

____

____

80. Is there any information you wish to add?

Please write below:

____

____

____

Thank you for your participation!

If you have any questions, please contact us via the contact form available on our web page: www.g2screening.se
