# Supplementary material for: How a population-based cohort of men estimate lifetime risk of prostate cancer in a survey before entering a prostate cancer screening trial in Sweden?
Source: BMJ Open. 2024 Aug 17;14(8):e083562. doi: 10.1136/bmjopen-2023-083562 (PMC11331866; doi:10.1136/bmjopen-2023-083562)
Supplement: online supplemental file 2 [file bmjopen-14-8-s002.pdf]

Supplementary document 2:

The assumptions of a linear regression with categorical independent variables are the following: a) No dependence of the residuals on predicted values b) Constant variance of residuals vs predicted values c) No pattern of residuals on predictor variables d) No pattern of residuals vs time (subject id) e) Normally distributed residuals (minor problem if large sample size and limited non-normality). In our case a) and e) are not fully satisfied, however due to the large sample size the violation of e) is not a large issue. The other assumptions are approximately satisfied. In order to investigate how the violations of the assumption affected the result, a sensitivity analysis was performed where data were analyzed after an arcsine square root transformation of the values of the perceived risks. After this transformation, a) is still not fully satisfied but the pattern is reduced, while the other assumptions are approximately satisfied. The results of the analyses on the original and transformed scale were very close, in particular the p-values and the estimates for significant variables. The results presented are based on the non-transformed data since interpretations of estimates based on transformed data is involved.
