# Supplementary material for: How a population-based cohort of men estimate lifetime risk of prostate cancer in a survey before entering a prostate cancer screening trial in Sweden?
Source: BMJ Open. 2024 Aug 17;14(8):e083562. doi: 10.1136/bmjopen-2023-083562 (PMC11331866; doi:10.1136/bmjopen-2023-083562)
Supplement: online supplemental file 3 [file bmjopen-14-8-s003.pdf]

Table S1. Background characteristics for attenders divided into subgroups based on risk estimation <15%, 15-25% >25% and their proportions for each characteristic.

Background characteristics		Total study population (13,189) 100%	Subgroups grouped by risk estimation and their proportions for each characteristic		
Perceived lifetime risk of prostate cancer			<15% (2903) 22%	15-25% (2543) 19%	>25% (7743) 59%
Previous prostate examination	Yes	7109	1356 19%	1308 18%	4445 63%
	No	5885	1503 26%	1199 20%	3183 54%
	Not available	195	44 23%	36 18%	115 59%
Family history of prostate cancer	Yes	1817	144 8%	200 11%	1473 81%
	No	7940	2200 28%	1715 22%	4025 51%
	Not available	3432	559 16%	628 18%	2245 65%
Physical exercise	Several times a week	5788	1383 24%	1161 20%	3244 56%
	Once a week	5272	1066 20%	1014 19%	3192 61%
	Never	1862	385 21%	318 17%	1159 62%
	Not available	267	69 26%	50 19%	148 55%
Healthy diet	Most commonly	8324	1995 24%	1640 20%	4689 56%
	Sometimes	4082	752 18%	755 18%	2575 63%
	Rarely	719	137 19%	136 19%	446 62%
	Not available	64	19 30%	12 19%	33 52%
Comorbidity	Yes	5306	1074 20%	983 19%	3249 61%
	No	7402	1738 23%	1465 20%	4199 57%
	Not available	481	91 19%	95 20%	295 61%
Current smoker	Yes	1204	248 21%	218 18%	738 61%
	No	11879	2633 22%	2309 19%	6937 58%
	Not available	106	22 21%	16 15%	68 64%

Alcohol consumption	I never drink alcohol	999	250<br>25%	166<br>17%	583<br>58%
	Normal	10740	2340<br>22%	2110<br>20%	6290<br>59%
	Risk consumption	1232	274<br>22%	231<br>19%	727<br>59%
	Not available	218	39<br>18%	36<br>17%	143<br>66%
Degree of education	Elementary school or equivalent	918	179<br>19%	152<br>17%	587<br>64%
	Upper secondary school or equivalent	6291	1299<br>21%	1178<br>19%	3814<br>61%
	University or college	5951	1416<br>24%	1209<br>20%	3326<br>56%
	Not available	29	9<br>31%	4<br>14%	16<br>55%
Partner	Has partner	10846	2388<br>22%	2106<br>19%	6352<br>59%
	No partner	2310	506<br>22%	433<br>19%	1371<br>59%
	Not available	33	9<br>27%	4<br>12%	20<br>61%
International prostate symptom score (IPSS)	No or mildly symptomatic	9716	2364<br>24%	1982<br>20%	5370<br>55%
	Moderately symptomatic	2346	346<br>15%	398<br>17%	1602<br>68%
	Severely symptomatic	369	39<br>11%	42<br>11%	288<br>78%
	Not available	758	154<br>20%	121<br>16%	483<br>64%
IIEF-5 estimation for erectile function	No erectile dysfunction	6816	1611<br>24%	1377<br>20%	3828<br>56%
	Mild erectile dysfunction	1606	282<br>18%	303<br>19%	1021<br>64%
	Mild to moderate erectile dysfunction	491	73<br>15%	65<br>13%	353<br>72%
	Moderate erectile dysfunction	174	24<br>14%	23<br>13%	127<br>73%
	Severe erectile dysfunction	129	17<br>13%	23<br>18%	89<br>69%
	No sexual activity has occurred	2787	593<br>21%	555<br>20%	1639<br>59%
	Not available	1186	303<br>26%	197<br>17%	686<br>58%
