# Supplementary material for: How a population-based cohort of men estimate lifetime risk of prostate cancer in a survey before entering a prostate cancer screening trial in Sweden?
Source: BMJ Open. 2024 Aug 17;14(8):e083562. doi: 10.1136/bmjopen-2023-083562 (PMC11331866; doi:10.1136/bmjopen-2023-083562)
Supplement: online supplemental file 4 [file bmjopen-14-8-s004.pdf]

Table S2. Factors analyzed for association to the risk estimation for the main study population (attenders).

Factors	Change in risk estimation	95% Confidence Interval		P-value
		Lower	Upper	
**Previous prostate examination**<br>No (reference)				
Yes	2.2	1.5	2.9	< 0.001
Not available	1.4	-1.4	4.2	0.34
**Family history of prostate cancer;**<br>No family history (reference)				
Yes	17.3	16.3	18.4	< 0.001
Not available	6.2	5.4	7.0	< 0.001
**Physical exercise;**<br>Several times a week (reference)				
Once a week	0.8	-0.0	1.5	0.04
Never	1.2	0.1	2.3	0.03
Not available	-2.5	-5.0	-0.1	0.05
**Healthy diet;**<br>Most commonly eating healthy diet (reference)				
Sometimes	2.4	1.6	3.1	< 0.001
Rarely	2.6	0.8	4.1	< 0.01
Not available	-0.4	-5.3	4.5	0.87
**Comorbidity;**<br>Not having comorbidity (reference)				
Yes	1.0	0.3	1.7	0.01
Not available	1.0	-0.8	2.9	0.27
**Smoking last month;**<br>No smoking (reference)				
Yes	1.1	-0.1	2.3	0.07
Not available	3.1	-0.7	6.9	0.11
**Alcohol consumption;**<br>No alcohol consumption (reference)				
Normal	0.3	-1.0	1.6	0.70
Risk consumption	0.5	-1.2	2.1	0.58
Not available	0.3	-2.6	3.2	0.85
**Degree of education;**<br>University or college (reference)				
Upper secondary school or equivalent	1.5	0.8	2.2	< 0.001
Elementary school or equivalent	2.1	0.7	3.5	< 0.01
Not available	1.5	-5.7	8.7	0.68
**Partner;**<br>Having partner (reference)				
No partner	0.2	-0.7	1.2	0.61
Not available	-0.6	-7.4	6.1	0.86
**International prostate symptom score (IPSS) for lower urinary tract symptoms;**				

No or mildly symptomatic (reference)				
Moderately symptomatic	5.6	4.7	6.5	< 0.001
Severely symptomatic	11.3	9.2	13.4	< 0.001
Not available	3.6	2.1	5.1	< 0.001
**IIEF-5 estimation of erectile function;**<br>No erectile dysfunction (reference)				
Mild erectile dysfunction	2.6	1.5	3.6	< 0.001
Mild to moderate erectile dysfunction	5.3	3.4	7.1	< 0.001
Moderate erectile dysfunction	3.5	0.5	6.5	0.02
Severe erectile dysfunction	2.5	-1.0	5.9	0.17
No sexual activity has occurred	0.2	-0.8	1.1	0.73
Not available	-0.7	-2.0	0.5	0.27
