# Supplementary material for: How a population-based cohort of men estimate lifetime risk of prostate cancer in a survey before entering a prostate cancer screening trial in Sweden?
Source: BMJ Open. 2024 Aug 17;14(8):e083562. doi: 10.1136/bmjopen-2023-083562 (PMC11331866; doi:10.1136/bmjopen-2023-083562)
Supplement: online supplemental file 5 [file bmjopen-14-8-s005.pdf]

Table S3. Factors analyzed for association to the risk estimation for non-attenders (men who answered the survey but did not take a PSA).

Factors	Change in risk estimation	95% Confidence Interval		P-value
		Lower	Upper	
**Previous prostate examination**<br>No (reference)				
Yes	3.5	1.1	5.9	0.01
Not available	-0.9	-9.7	7.9	0.84
**Family history of prostate cancer;**<br>No family history (reference)				
Yes	16.2	12.5	19.8	< 0.001
Not available	8.9	6.1	11.6	< 0.001
**Physical exercise;**<br>Several times a week (reference)				
Once a week	-0.3	-3.0	2.4	0.85
Never	1.6	-2.0	5.2	0.38
Not available	-3.3	-11.6	5.0	0.43
**Healthy diet;**<br>Most commonly eating healthy diet (reference)				
Sometimes	3.0	0.4	5.7	0.02
Rarely	3.6	-1.0	8.1	0.12
Not available	1.9	-10.6	14.3	0.77
**Comorbidity;**<br>Not having comorbidity (reference)				
Yes	2.4	-0.1	4.9	0.06
Not available	1.8	-3.9	7.5	0.54
**Smoking last month;**<br>No smoking (reference)				
Yes	0.1	-3.1	3.3	0.94
Not available	-3.6	-12.4	5.2	0.42
**Alcohol consumption;**<br>No alcohol consumption (reference)				
Normal	-0.5	-4.4	3.3	0.78
Risk consumption	0.0	-4.9	4.9	0.99
Not available	-2.3	-10.6	6.0	0.58
**Degree of education;**<br>University or college (reference)				
Upper secondary school or equivalent	-0.2	-2.7	2.3	0.86
Elementary school or equivalent	5.4	0.7	10.1	0.02
Not available	-1.1	-17.1	15.0	0.90
**Partner;**<br>Having partner (reference)				
No partner	0.5	-2.5	3.4	0.76
Not available	-1.2	-14.6	12.2	0.86

**International prostate symptom score (IPSS) for lower urinary tract symptoms;**<br>No or mildly symptomatic (reference)				
Moderately symptomatic	2.4	-1.0	5.8	0.16
Severely symptomatic	10.4	3.8	17.0	< 0.01
Not available	4.7	0.0	9.3	0.05
**IIEF-5 estimation of erectile function;**<br>No erectile dysfunction (reference)				
Mild erectile dysfunction	0.3	-3.5	4.1	0.87
Mild to moderate erectile dysfunction	0.8	-4.5	6.0	0.78
Moderate erectile dysfunction	1.1	-7.8	10.1	0.80
Severe erectile dysfunction	4.7	-9.2	18.6	0.51
No sexual activity has occurred	-1.4	-4.7	1.8	0.39
Not available	-1.1	-5.2	3.1	0.62
