# Supplementary material for: How a population-based cohort of men estimate lifetime risk of prostate cancer in a survey before entering a prostate cancer screening trial in Sweden?
Source: BMJ Open. 2024 Aug 17;14(8):e083562. doi: 10.1136/bmjopen-2023-083562 (PMC11331866; doi:10.1136/bmjopen-2023-083562)
Supplement: Supplementary file 6 [file bmjopen-2023-083562.reviewer_comments.pdf]

PEER REVIEW HISTORY

BMJ Open publishes all reviews undertaken for accepted manuscripts. Reviewers are asked to complete a checklist review form (**http://bmjopen.bmj.com/site/about/resources/checklist.pdf**) and are provided with free text boxes to elaborate on their assessment. These free text comments are reproduced below.

ARTICLE DETAILS

**TITLE (PROVISIONAL)**	How a population based cohort of men estimate lifetime risk of prostate cancer in a survey before entering a prostate cancer screening trial in Sweden
**AUTHORS**	Palmstedt, Emmeli; Månsson, Marianne; Kollberg, Karin Stinesen; Carlsson, Sigrid; Hellström, Mikael; Wallström, Jonas; Hugosson, Jonas; Arnsrud Godtman, Rebecka

VERSION 1 – REVIEW

**REVIEWER**	Katy Bell<br>The University of Sydney Faculty of Medicine and Health, School of Public Health
**REVIEW RETURNED**	27-Jan-2024

**GENERAL COMMENTS**	Thank you for the opportunity to review this report of a survey on men's perceived risk of prostate cancer when invited to participate in a screening trial. The findings show that men appear to overestimate risk, and thereby potential benefit of screening, and point to the need for more informed decisions when considering whether or not to undergo screening. I have some suggestions for how it might be improved.<br>1. Abstract. Include calendar years that survey question was asked Abstract and main paper). Provide brief explanation for why lifetime risk was anticipated to be 20% (needs to be in Abstract as well as full paper).<br>2. Page 4, 54-58: "Being provided adequate information is important for men to make this informed decision. A key component is having a realistic assessment of the lifetime risk of being diagnosed with prostate cancer." There is known to be substantial overdiagnosis associated with prostate cancer screening which will inflate the lifetime risk of being diagnosed with cancer. Knowledge of lifetime risk of dying from prostate cancer is more important for informed decision making about screening. This should be provided along with potential benefits and harms of screening, ideally with graphics like icon arrays to support understanding of risk and informed decisions.<br>3. Methods. Were men in the control arm also surveyed about perceived cancer risk, or only those in the intervention arm. This needs to be made clearer<br>4. Provide the full survey questions as a supplement. How was family history defined?<br>5. Page 5, lines 7-12: ""During your lifetime, how high do you estimate that your risk of being<br>diagnosed with prostate cancer is?" Men were asked to estimate their lifetime risk by putting a mark on a visual analog scale from 0% to 100%, where a number also was displayed while marking". Were any other questions to see how well they understood this metric?

	Were iconarrays or other graphics used to help communicate risk? Were other examples of lifetime risk given? Risk is known to be a difficult concept for many people, and many of the participants may not have understood the question being asked. If this was the only question/material related to risk in the survey, you need to include this as a limitation.<br>6. Present non-attendees in the main section, and include same level of detail as for attendees. Present distributions of participant estimate – anticipated estimate (20%) (separately for attendees and non-attendees). Likewise for participant estimate – 12% (risk of symptomatic cancer – i.e. doesn't include overdiagnosed cancers).<br>7. From the box plots presented, the data appear skewed for both attendees and non-attendees. Consider applying a transformation such as logit so that data are distributed as real numbers rather than probability (which is bound by 0,1), and use these in the regression model.<br>8. The distributions appear to be different for attendees and non-attendees. Can you test for a difference in distributions and not just in medians/means? Can also perform standard tests after applying the logit transformation to see if there is a difference in means.<br>9. State the assumptions of the regression model, and how well your data met these assumptions.<br>10. Specify whether the potential confounders described in the DAG were all included in the model at once, or if different sets were included in different models<br>11. Discussion. "The lifetime risk of symptomatic prostate cancer in Sweden is approximately 13%": provide a reference for where this estimate is from. You may wish to note that this estimate is similar to an Australian lifetime risk estimate of 11.5% for risk in 1982 (before PSA screening) adjusted for improved longevity:<br>https://bmjopen.bmj.com/content/9/3/e022457.abstract<br>12. "We can only speculate why men in our study overestimated their risk of prostate cancer.<br>Possible explanations could be public campaigns aiming at raising awareness about prostate<br>cancer." Another explanation may be that the trialists information in the invitation letter also implied high risk of prostate cancer: "prostate cancer is the most common cancer among Swedish men and also the most common cancer-related cause of death".<br>13. Have the trialists made attempts to provide more accurate risk information to the trial participants? Will men who completed the survey be sent a plain language summary of the current report? Are there any plans for qualitative study (e.g. participant interviews) to investigate possible reasons for the overestimation of risk? Such a study could also explore impact of perceived risk, and why non-attendees who also overestimated risk decided not to have screening.<br>14. You could add that findings are in line with evidence showing patients and doctors in general overestimate benefits and underestimate harms of medical interventions:<br>https://jamanetwork.com/journals/jamainternalmedicine/article-abstract/2038981<br>https://jamanetwork.com/journals/jamainternalmedicine/article-abstract/2596010<br>15. "Our findings show that there is an urgent need to improve information concerning prostate<br>cancer risk so that men can make a well-informed decision on whether to be screened for<br>prostate cancer or not. We agree with the proposal by Gnanapragasam and colleagues who suggest that the link between

	urinary symptoms and prostate cancer should be removed (31) and the public messaging should instead emphasize that prostate cancer commonly is silent or asymptomatic, particularly in the curable stages of the disease". A priority for well-informed decisions is to include accurate information on potential benefits and harms of prostate cancer screening. Risk of overdiagnosis and overtreatment must be communicated alongside potential benefits, and numerical information including graphical display such as icon arrays should be used according to best practice for developing decision aids. https://journals.sagepub.com/doi/10.1177/23814683221129875

**REVIEWER**	Yiwen Zhang<br>Harvard University T H Chan School of Public Health
**REVIEW RETURNED**	14-Apr-2024

**GENERAL COMMENTS**	This manuscript presented an interesting topic on how men perceive their lifetime risk of prostate cancer and examined factors associated with the estimation. The study design and method is clear and appropriate. The discussion is comprehensive.<br><br>I have one comment that would like to hear the authors' thoughts: does the 'overestimation' really overestimate? In real life, assuming the lifetime risk of prostate cancer for every man is the same (20% used in this paper) is a bit unreasonable, since we already know (and the patients may also know) that the risk of prostate cancer is different based on each man's characteristic and lifestyle factors. If a man with a family history and have bad lifestyle correctly perceives his lifetime risk of prostate cancer as higher above the average, like 35%, that's probably not over-estimating, it actually reflects the fact that this man is aware he is at a higher risk of prostate cancer. It would be interesting if the authors could use different average risks for men with different lifetime risks, like a higher-risk population and a low-risk population, and see how these two groups may perceive their risk differently.<br><br>Minor points:<br>1. In Table 1, it would be interesting to also show the percentage of men perceived higher risk vs. lower risk of prostate cancer.<br>2. In the multivariable analysis corresponding to Figure 4, were all the factors put into the same model? Does the model adjust for age?

**REVIEWER**	Zhengyi Deng<br>Stanford University School of Medicine
**REVIEW RETURNED**	22-Apr-2024

**GENERAL COMMENTS**	In this study, the authors compared the perceived lifetime risk of prostate cancer and observed lifetime risk of prostate cancer among male participants in the screening arm of the GÖTEBORG-2 trial. They found the median of the perceived risk was 30%, which was statistically significantly higher than the observed risk of 20%. Also, they evaluated the association of factors associated with the perceived risk estimation. There are major limitations in this study, which requires significant revisions to improve the work.<br>1. In the secondary analyses, the authors defined their outcome as proportion of men who estimated their lifetime risk as <15% or >25%. My understanding is that people who had the risk <15% or >25% were coded as 1 and others were coded as 0, which was a binary outcome. In this case, a linear regression is not appropriate, as this model is for continuous outcome. In addition, it is even more

	confusing that the results in Figure 4 and Table S2 provide estimates in percentage. Therefore, it is very confusing what method was used for this analysis and whether the results were valid and interpreted in a correct way. The authors need to clarify the outcome definition and provide a equation of the model.<br><br>2. The authors mentioned that a DAG was constructed to pre-specify the anticipated associations between predictor and the outcome variable. I'm not sure why a DAG was used here. DAG is often used to delineate causations between exposure and outcome, and to identify potential confounders to be accounted for. However, their secondary analysis aimed to evaluate the association between many factors and the estimated risk, which was a risk factor analysis rather than a causal analysis.<br><br>3. The interpretations of the results from secondary analysis are questionable. Please refer to comment #1 to clarify or revise the methods and results.<br><br>4. The authors mentioned that "family history of prostate cancer, LUTS, and ED which were associated with >5 percentage points higher risk estimation." However, based on the methods, outcome was defined as proportion of men who estimated their lifetime risk as <15% or >25%. So, these factors should be associated with either >5% higher or lower risk. As you can see, the outcome definition makes the interpretation of the results really hard.<br><br>5. In discussion, the authors mentioned that "These men may decide to have a PSA-test based on the assumption that they are at risk for prostate cancer, while those without urinary dysfunction may abstain from testing themselves in a false sense of security, believing that they are not at risk for prostate cancer, potentially leading to a delayed diagnosis." This sentence opens a question that what to do to improve their perception of their prostate cancer risk. I think the discussion lacks relevant information on strategies to improve perception, and the authors should talk more about that. For example, a prostate cancer risk prediction model might yield a more accurate risk prediction.

VERSION 1 – AUTHOR RESPONSE

Reviewer: 1
Dr. Katy Bell, The University of Sydney Faculty of Medicine and Health
Comments to the Author:
Thank you for the opportunity to review this report of a survey on men's perceived risk of prostate cancer when invited to participate in a screening trial. The findings show that men appear to overestimate risk, and thereby potential benefit of screening, and point to the need for more informed decisions when considering whether or not to undergo screening. I have some suggestions for how it might be improved.

1. Abstract. Include calendar years that survey question was asked Abstract and main paper). Provide brief explanation for why lifetime risk was anticipated to be 20% (needs to be in Abstract as well as full paper).

- Reply: Calendar years is now added in the abstract as well as an explanation to the 20%, the sentence now reads:

"The primary outcome was the median lifetime risk estimation, which was compared with Wilcoxon test to an anticipated lifetime risk of 20% (based on GÖTEBORG-1 trial)."

2. Page 4, 54-58: “Being provided adequate information is important for men to make this informed decision. A key component is having a realistic assessment of the lifetime risk of being diagnosed with prostate cancer.” There is known to be substantial overdiagnosis associated with prostate cancer screening which will inflate the lifetime risk of being diagnosed with cancer. Knowledge of lifetime risk of dying from prostate cancer is more important for informed decision making about screening. This should be provided along with potential benefits and harms of screening, ideally with graphics like icon arrays to support understanding of risk and informed decisions.

- Reply: We agree that it is important that men have realistic knowledge about their lifetime risk of dying from prostate cancer for making a balanced informed decision. In the information to men, we provided them information on risk of prostate cancer death but no numerical numbers. See page 3 in the manuscript: “prostate cancer is the most common cancer among Swedish men and also the most common cancer-related cause of death”.
  In retrospect, we could however have provided more detailed risk estimation as the reviewer suggested. Using graphic icons is a good suggestion for improving future surveys. Since this study has already been completed, we cannot change its appearance.

3. Methods. Were men in the control arm also surveyed about perceived cancer risk, or only those in the intervention arm. This needs to be made clearer

- Reply: Men in the control arm were not asked to answer the survey which is clarified in the method section on page 3:

“Men aged 50 to 60 years were randomized into two groups, one invited to a PSA+MRI-based screening and one constituting a control group that was neither invited to the screening nor to the survey (2:1 ratio).”

4. Provide the full survey questions as a supplement. How was family history defined?

- Reply: The full survey questions are now added as supplement. Family history was defined as any relative who had prostate cancer.

5. Page 5, lines 7-12: ““During your lifetime, how high do you estimate that your risk of being diagnosed with prostate cancer is?” Men were asked to estimate their lifetime risk by putting a mark on a visual analog scale from 0% to 100%, where a number also was displayed while marking”. Were any other questions to see how well they understood this metric? Were iconarrays or other graphics used to help communicate risk? Were other examples of lifetime risk given? Risk is known to be a difficult concept for many people, and many of the participants may not have understood the question being asked. If this was the only question/material related to risk in the survey, you need to include this as a limitation.

- Reply: This was the only question on risk estimation in the survey. It is clarified in the discussion section and added as a limitation on page 7-8.

“Risk assessment is for many people a difficult concept. A limitation of this study is therefore that the risk assessment was done only by a one-item numerical rating scale from 0-100%. In previous studies in breast cancer, this type of measurement often resulted in overestimating of risk, because the anchors were sometimes misleading, and some women perceived their risk of developing breast cancer as 50%, not understanding that this means a 1 in 2 chance (26). However, we acknowledge that there is no gold standard measurement for perceived risk and the one-item numerical rating scale is a validated instrument with acceptable psychometric properties that outperforms other items and format and is frequently used (27, 28).

There were no other questions regarding how well men understood the concept of lifetime risk.”

6. Present non-attendees in the main section, and include same level of detail as for attendees. Present distributions of participant estimate – anticipated estimate (20%) (separately for attendees

and non-attendees). Likewise for participant estimate – 12% (risk of symptomatic cancer – i.e. doesn't include overdiagnosed cancers).

- Reply: Table S1 changed to Table 2 and was moved from supplementary to the main text so that background characteristics for non-attenders are in the main section. We have also provided a forest plot figure (figure 5) for this group and added following in the text, results section on page 6:
  "Of the 38,775 randomized men, 1264 men answered the survey but chose not to have a PSA-test (non-attenders). These men also estimated their lifetime risk to a median 30% (IQR 14-50), significantly higher than the anticipated risk of 20% (p < 0.001) (Figure 3). Additional information regarding non-attenders is presented in Table 2. The percentage of men estimating their risk to be lower than 15% were 25.8% (326/1264), 14.5% (183/1264) had a risk estimation between 15-25% and 59.7% (755/1264) estimated their risk to be above 25%.

  In the multivariable analysis for non-attenders, factors with a statistically significant association to lifetime risk estimation were: prior prostate examination, family history of prostate cancer, sometimes healthy diet compared to most commonly healthy diet, lower level of education than university or college, severe LUTS (Figure 5). Factors associated with >5 percentage points higher risk estimation were family history of prostate cancer, lower level of education than university or college and severe LUTS. Results are shown in Supplementary Table S3."

  And added in the abstract:
  "Participants – 17,980 men participated in PSA-testing of whom 13,189 answered the survey. In addition, 1264 men answered the survey only."

  Following was also added in the discussion section on page 7:
  "Interestingly, non-attenders' risk estimation was almost the same as attenders'. The median and upper quartile are identical, while more men among attenders predicted their risk low than among non-attenders as can be seen in Figure 3. In the linear regression, the statistically significant factors for non-attenders were also significant for attenders. However, more factors were non-significant for non-attenders. This is partly due to the smaller sample size leading to lower power. For instance, the point estimates for comorbidity were higher for non-attenders than for attenders, although not statistically significant. Altogether, the results show that risk estimation among attenders and non-attenders are similar, indicating that these results can be generalized to men of the same age and background."

  And as to det latter part of the comment we have added a line in figure 3 at 12% to mark the anticipated risk of having symptomatic cancer.
  If we have understood your comment correctly, you would like boxplots similar to these below. We have a concern that these figures will not add additional value to the article and could perhaps even be confusing for some. Therefore, we have decided not to use them in the article.

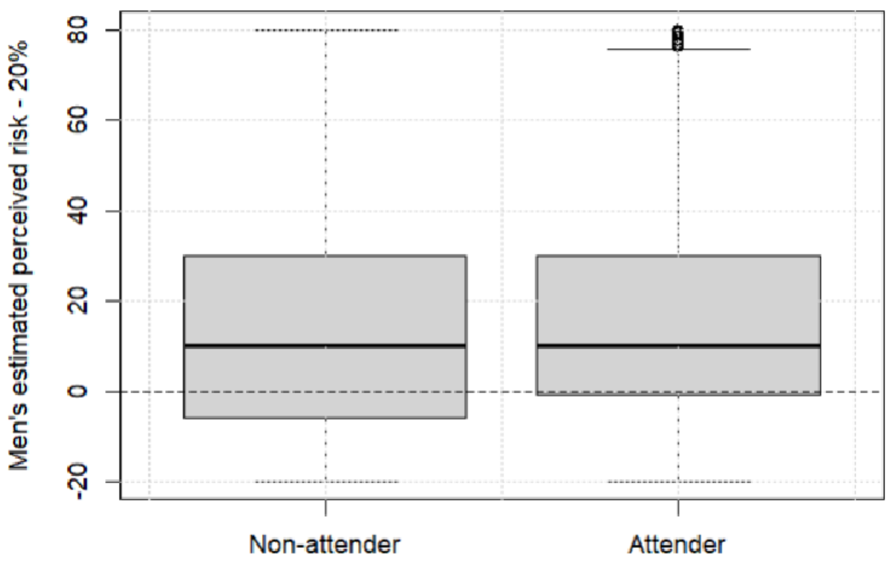

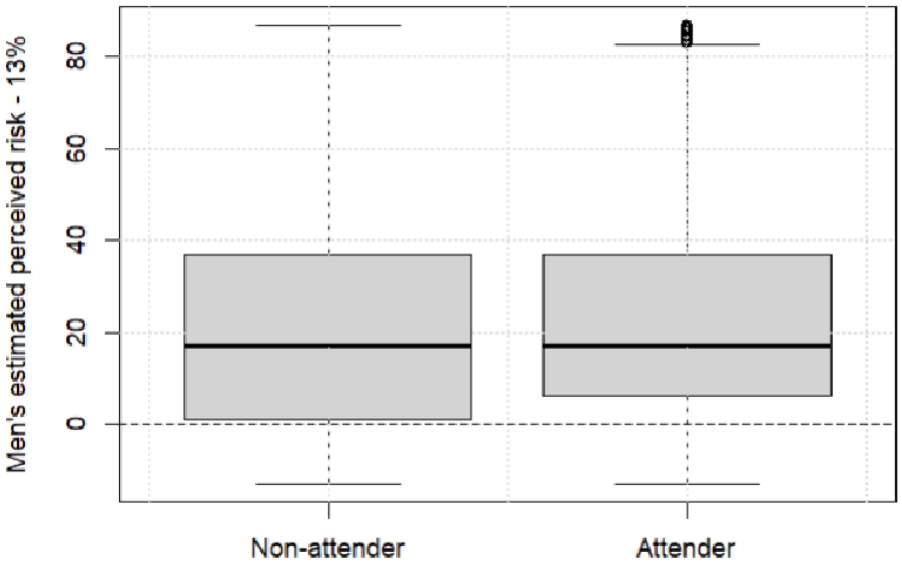

7. From the box plots presented, the data appear skewed for both attendees and non-attendees. Consider applying a transformation such as logit so that data are distributed as real numbers rather than probability (which is bound by 0,1), and use these in the regression model.

- Reply: The distributions are certainly somewhat skewed and to use a transform might be a good idea. We were aware of that the assumptions of a linear regression on the original scale were not fully met, but chose to do the analysis on the original scale anyhow for some reasons: Firstly, the bulk of the predictions aren't close to neither 0 nor 1 and the skewness isn't that severe, secondly the data set is so large so that the non-normality of residuals does not have a large impact, thirdly to avoid having estimates that are difficult to interpret, and finally that the results on the original scale are robust compared to using transformed data. The conditions which are not satisfied are that the residuals aren't normally distributed and that there is a pattern in the residuals, the other assumptions are at least close to satisfied. The non-normality is hardly a problem since the skewness isn't that severe, and we have very a large data set.

It is difficult to find a transformation of our data which satisfies all assumptions, but the arcsine transform (arcsine(sqrt(y)) appears to be best among the ones considered (logit, sqrt, box-cox, arcsine). The residuals become closer to normal, and the pattern in the residuals reduced.
In the table below, p-values are presented for both the original and transformed data. They are mostly similar, in particular they are on the same side of the 0.05 cut-off except in one case (p=0.025 and 0.051 in the original and transformed data, respectively; never exercise vs exercise several times a week). The estimates from original and transformed data are not possible to directly compare. In the transformed case it is possible to back-transform estimates however, they depend on the value of the perceived risk for a certain combination of all other variables except the one of interest. In the table below the estimates are given for combinations of the other variables which corresponds to the median perceived risk (30%), and they are then relatively close to the original ones, in particular for the significant variables. See this table:

	Est_orig	pValue_orig	Est_arcsine_median	pValue_arcsine
Earlier prostate exam (yes vs no)	2.2	0.000	2.5	0.000
Earlier prostate exam (NA vs no)	1.4	0.347	1.6	0.304
Family history of PC (yes vs no)	17.3	0.000	19.3	0.000
Family history of PC (NA vs no)	6.2	0.000	6.9	0.000
Phys. exercise (one vs many times/wk)	0.8	0.039	0.9	0.035
Phys. exercise (none vs many times/wk)	1.2	0.025	1.2	0.051
Phys. exercise (NA vs many times/wk)	-2.5	0.046	-2.7	0.048
Healthy diet (sometimes vs most commonly)	2.4	0.000	2.8	0.000
Healthy diet (rarely vs most commonly)	2.6	0.001	2.8	0.002
Healthy diet (NA vs most commonly)	-0.4	0.869	-0.9	0.745
Comorbidity (yes vs no)	1.0	0.005	1.0	0.009
Comorbidity (NA vs no)	1.0	0.268	1.0	0.321
Alcohol consumption (no risk vs none)	0.3	0.700	0.8	0.297
Alcohol consumption (risk vs none)	0.5	0.587	1.1	0.248
Alcohol consumption (NA vs none)	0.3	0.865	0.8	0.601
Smoking (yes vs no)	1.1	0.070	1.1	0.108
Smoking (NA vs no)	3.4	0.054	3.6	0.068
Educ. (upper 2ndary etc vs university)	1.5	0.000	1.5	0.000
Educ. (elementary etc vs university)	2.1	0.003	2.1	0.008
Educ. (NA vs university)	1.5	0.682	0.0	1.000
Partner (No partner vs having partner)	0.2	0.603	0.2	0.763
Partner (NA vs having partner)	-0.6	0.858	-1.9	0.618
Urinary symp. (medium vs none/low)	5.6	0.000	6.2	0.000
Urinary symp. (severe vs none/low)	11.3	0.000	12.3	0.000
Urinary symp. (NA vs none/low)	3.6	0.000	3.5	0.000
Erectile dysf. (mild vs none)	2.6	0.000	2.9	0.000
Erectile dysf. (mild to mod. vs none)	5.3	0.000	5.5	0.000
Erectile dysf. (moderate vs none)	3.5	0.021	3.8	0.023
Erectile dysf. (severe vs none)	2.5	0.163	2.9	0.129
Erectile dysf. (no sexual activity vs none)	0.2	0.726	0.3	0.567
Erectile dysf. (NA vs none)	-0.7	0.261	-1.1	0.127

For these reasons we would like to stay with the results based on the original data. However, we will discuss assumptions in the manuscript, as suggested by the reviewer in bullet point 9). Furthermore, we will add to the manuscript that a sensitivity analysis on transformed data was performed, that results were close, and that the estimates are approximative as the assumptions aren't fully satisfied. The following text is added to the manuscript in statistical methods page 5:
"Note that the estimates and p-values are approximate since not all assumptions of a linear regression are fully satisfied. The assumptions of the regression and a sensitivity analysis based on transformed data, which resulted in very similar results, are discussed in the Supplementary document 1."

This is added in supplement as supplement document:
"The assumptions of a linear regression with categorical independent variables are the following: a) No dependence of the residuals on predicted values b) Constant variance of residuals vs predicted values c) No pattern of residuals on predictor variables d) No pattern of

residuals vs time (subject id) e) Normally distributed residuals (minor problem if large sample size and limited non-normality). In our case a) and e) are not fully satisfied, however due to the large sample size the violation of e) is not a large issue. The other assumptions are approximately satisfied. In order to investigate how the violations of the assumption affected the result, a sensitivity analysis was performed where data were analyzed after an arcsine square root transformation of the values of the perceived risks. After this transformation, a) is still not fully satisfied but the pattern is reduced, while the other assumptions are approximately satisfied. The results of the analyses on the original and transformed scale were very close, in particular the p-values and the estimates for significant variables. The results presented are based on the non-transformed data since interpretations of estimates based on transformed data is involved."

8. The distributions appear to be different for attendees and non-attendees. Can you test for a difference in distributions and not just in medians/means?

– Reply: When testing for a difference between distributions using the Kolmogorov-Smirnov test the p-value is 0.05, hence there is a statistically significant difference between the distributions. In this case the median and IQR for the two distributions are:
  Attender: 30 (14-50)
  Non-attender: 30 (19-50)
  Hence the difference is in the lower part of the distributions with more low values among attenders. We prefer not to add the statistical test to the manuscript, since it was not part of the statistical plan and performed after data was viewed, but we added the following text to the manuscript on page 7:
  "The median and upper quartile are identical, while more men among attenders predicted their risk low than among non-attenders as can be seen in Figure 3."

Can also perform standard tests after applying the logit transformation to see if there is a difference in means.

– Reply: Due to the large number of men, a standard t-test can be used on the original data to test difference in means. The mean in both groups is 33.6, and the difference in mean is 0 (95% CI -1.3, 1.3), p>0.99. We prefer not to add the statistical test to the manuscript, since it was not part of the statistical plan and performed after data was viewed.

9. State the assumptions of the regression model, and how well your data met these assumptions.

– Reply: We thank the reviewer for this suggestion, and have added this to the manuscript, see answer to bullet point 7.

10. Specify whether the potential confounders described in the DAG were all included in the model at once, or if different sets were included in different models

– Reply: The DAG was used only as discussion material giving an overview on how different factors were connected to each other when selecting which factors to choose for analyzing. We have chosen to remove the sentence concerning DAG because of the risk of misinterpretation. Also read the answer to the third reviewer at bullet point 2.

11. Discussion. "The lifetime risk of symptomatic prostate cancer in Sweden is approximately 13%": provide a reference for where this estimate is from. You may wish to note that this estimate is similar to an Australian lifetime risk estimate of 11.5% for risk in 1982 (before PSA screening) adjusted for improved longevity: https://bmjopen.bmj.com/content/9/3/e022457.abstract

– Reply: We thank the reviewer for contributing with this reference. We have added this reference together with two others and slightly changed the sentence, see page 8:

"The lifetime risk of symptomatic prostate cancer before the PSA-era has been estimated to approximately 11-13% which means that if men interpreted the question as lifetime risk of symptomatic prostate cancer, the overestimation was even larger (Figure 3)"

12. "We can only speculate why men in our study overestimated their risk of prostate cancer. Possible explanations could be public campaigns aiming at raising awareness about prostate cancer." Another explanation may be that the trialists information in the invitation letter also implied high risk of prostate cancer: "prostate cancer is the most common cancer among Swedish men and also the most common cancer-related cause of death".

- Reply: Good point, this is added in the discussion section, page 8.

"Public campaigns aiming at raising awareness about prostate cancer and the invitation letter mentioning "prostate cancer is the most common cancer among Swedish men" may influence men´s perception."

13. Have the trialists made attempts to provide more accurate risk information to the trial participants? Will men who completed the survey be sent a plain language summary of the current report? Are there any plans for qualitative study (e.g. participant interviews) to investigate possible reasons for the overestimation of risk? Such a study could also explore impact of perceived risk, and why non-attendees who also overestimated risk decided not to have screening.

- Reply: This is a very important subject, how to improve information to men so that they can make a balanced shared decision on screening participation. We are currently discussing how we should improve information to men who are invited to our ongoing screening trial. After publication of this study, we plan to present a lay person summary at the trial website from this study.
  There are no plans for a qualitative study, but it is a good idea for future research.

14. You could add that findings are in line with evidence showing patients and doctors in general overestimate benefits and underestimate harms of medical interventions:
https://jamanetwork.com/journals/jamainternalmedicine/article-abstract/2038981
https://jamanetwork.com/journals/jamainternalmedicine/article-abstract/2596010

- Reply: Thank you for these suggestions, we have rewritten one sentence in the discussion and added these references on page 9:
  "Our findings indicate that men overestimate the benefit of screening which is in line with other reports showing that both patients and healthcare workers tend to overestimate the benefits from medical interventions (40, 41)."

15. "Our findings show that there is an urgent need to improve information concerning prostate cancer risk so that men can make a well-informed decision on whether to be screened for prostate cancer or not. We agree with the proposal by Gnanapragasam and colleagues who suggest that the link between urinary symptoms and prostate cancer should be removed (31) and the public messaging should instead emphasize that prostate cancer commonly is silent or asymptomatic, particularly in the curable stages of the disease". A priority for well-informed decisions is to include accurate information on potential benefits and harms of prostate cancer screening. Risk of overdiagnosis and overtreatment must be communicated alongside potential benefits, and numerical information including graphical display such as icon arrays should be used according to best practice for developing decision aids. https://journals.sagepub.com/doi/10.1177/23814683221129875

- Reply: Thank you for the reference and we agree that the risk of overdiagnosis and overtreatment should be included in the information for men to be able to make an informed decision. We have added this in the following sentences and added the reference on page 9:

“ *Thus, there is a need to improve information concerning prostate cancer risk, risk of overdiagnosis and outcomes after medical interventions helping men to get a realistic risk perception. Prediction models may help to obtain personalized risk estimates. Such improved information could be communicated using icon arrays and numerical risk estimates, so that men can make a well-informed decision on whether to be screened or not for prostate cancer (42). Urinary symptoms is a common reason why men ask for PSA-testing but the link between urinary symptoms and prostate cancer should be removed (33, 37) and the public messaging should instead emphasize that prostate cancer commonly is silent or asymptomatic, particularly in the curable stages of the disease. Men with urinary symptoms should have the same information regarding benefits and harms of screening for prostate cancer.”*

Reviewer: 2
Dr. Yiwen Zhang, Harvard University T H Chan School of Public Health
Comments to the Author:
This manuscript presented an interesting topic on how men perceive their lifetime risk of prostate cancer and examined factors associated with the estimation. The study design and method is clear and appropriate. The discussion is comprehensive.
I have one comment that would like to hear the authors' thoughts: does the 'overestimation' really overestimate? In real life, assuming the lifetime risk of prostate cancer for every man is the same (20% used in this paper) is a bit unreasonable, since we already know (and the patients may also know) that the risk of prostate cancer is different based on each man's characteristic and lifestyle factors. If a man with a family history and have bad lifestyle correctly perceives his lifetime risk of prostate cancer as higher above the average, like 35%, that's probably not over-estimating, it actually reflects the fact that this man is aware he is at a higher risk of prostate cancer. It would be interesting if the authors could use different average risks for men with different lifetime risks, like a higher-risk population and a low-risk population, and see how these two groups may perceive their risk differently.

- Reply: The strongest risk factors for prostate cancer are age, family history and geographical place of living. In Sweden, the lifetime risk of prostate cancer is mainly dependent on family history and life shortening comorbidity. As pointed out in table S2 men with family history report higher risk estimates and is mentioned in the discussion section on page 8. Men with comorbidity report a slightly higher prostate cancer risk estimation compared to men not having comorbidity. We have decided not to discuss these subgroup variations more extensive than the results displayed in table S2 and the discussion part concerning family history.

Minor points:
1. In Table 1, it would be interesting to also show the percentage of men perceived higher risk vs. lower risk of prostate cancer.
- Reply: In supplement we have added the table S1 which present background characteristic according to risk estimation percentages. A sentence in the result section is added on page 5-6:
"Information on background characteristics for subgroups according to risk estimation (below 15%, 15-25% and above 25%) are shown in Supplementary Table S1."

2. In the multivariable analysis corresponding to Figure 4, were all the factors put into the same model? Does the model adjust for age?

- Reply: Yes all factors were put into the same model and were not adjusted for age.

Reviewer: 3
Dr. Zhengyi Deng, Stanford University School of Medicine
Comments to the Author:
In this study, the authors compared the perceived lifetime risk of prostate cancer and observed lifetime risk of prostate cancer among male participants in the screening arm of the GÖTEBORG-2 trial. They found the median of the perceived risk was 30%, which was statistically significantly higher than the observed risk of 20%. Also, they evaluated the association of factors associated with the perceived risk estimation. There are major limitations in this study, which requires significant revisions to improve the work.

1. In the secondary analyses, the authors defined their outcome as proportion of men who estimated their lifetime risk as <15% or >25%. My understanding is that people who had the risk <15% or >25% were coded as 1 and others were coded as 0, which was a binary outcome. In this case, a linear

regression is not appropriate, as this model is for continuous outcome. In addition, it is even more confusing that the results in Figure 4 and Table S2 provide estimates in percentage. Therefore, it is very confusing what method was used for this analysis and whether the results were valid and interpreted in a correct way. The authors need to clarify the outcome definition and provide a equation of the model.

- Reply: Perhaps a misunderstanding is the reason for finding it confusing. We agree with you that the method, multivariable linear regression, should be used for continuous data and not binary. In this case we have used continuous data as the participating men estimate their risk on a scale from 0-100%. The sentence with secondary analyses may have been misunderstood and interpreted as that the secondary analyses were performed on the groups estimating <15% and >25% instead of being two different secondary analyses. In the first, we calculated the proportion of men estimating their risk <15%, between 15% and 25% or >25%. In the second analysis, factors that influenced the risk estimation were analyzed. For the risk factors a multivariable linear regression model with data from the entire population, no subgroups, was used. A clarification with changes in the text have been done, see page 5. *"There were two secondary analyses. In the first, we calculated the proportion of men who estimated their lifetime risk as < 15%, between 15% and 25% and > 25%. In the second, the association between various factors and men's perceived lifetime risk was evaluated using linear regression."*

2.The authors mentioned that a DAG was constructed to pre-specify the anticipated associations between predictor and the outcome variable. I'm not sure why a DAG was used here. DAG is often used to delineate causations between exposure and outcome, and to identify potential confounders to be accounted for. However, their secondary analysis aimed to evaluate the association between many factors and the estimated risk, which was a risk factor analysis rather than a causal analysis.

- Reply: We agree on that it is a risk factor analysis and not a causal analysis. It needs to be more specified that the DAG was only used as discussion material with an overview on how different factors were connected to each other when selecting which factors to use for analyzing. Because this can be misinterpreted and no effect on the reproducibility for future studies if being removed, we have decided to remove the sentence concerning DAG.

3. The interpretations of the results from secondary analysis are questionable. Please refer to comment #1 to clarify or revise the methods and results.

- Reply: see reply on comment #1.

4. The authors mentioned that "family history of prostate cancer, LUTS, and ED which were associated with >5 percentage points higher risk estimation." However, based on the methods, outcome was defined as proportion of men who estimated their lifetime risk as <15% or >25%. So, these factors should be associated with either >5% higher or lower risk. As you can see, the outcome definition makes the interpretation of the results really hard.

- Reply: These risk factors are calculated with a multivariable linear regression model for all men and not divided into subgroups based on estimating their risk <15%, 15-25% and >25%. Please read the reply on comment #1.

5. In discussion, the authors mentioned that "These men may decide to have a PSA-test based on the assumption that they are at risk for prostate cancer, while those without urinary dysfunction may abstain from testing themselves in a false sense of security, believing that they are not at risk for prostate cancer, potentially leading to a delayed diagnosis." This sentence opens a question that what to do to improve their perception of their prostate cancer risk. I think the discussion lacks relevant information on strategies to improve perception, and the authors should talk more about that. For example, a prostate cancer risk prediction model might yield a more accurate risk prediction.

- Reply: It is a thoughtful suggestion and we have therefore added the following to the discussion on page 9:
  "Our findings indicate that men overestimate the benefit of screening which is in line with other reports showing that both patients and healthcare workers tend to overestimate the benefits from medical interventions (40, 41). Thus, there is a need to improve information concerning prostate cancer risk, risk of overdiagnosis and outcomes after medical interventions helping men to get a realistic risk perception. Prediction models may help to obtain personalized risk estimates. Such improved information could be communicated using icon arrays and numerical risk estimates, so that men can make a well-informed decision on whether to be screened or not for prostate cancer (42). Urinary symptoms is a common reason why men ask for PSA-testing but the link between urinary symptoms and prostate cancer should be removed (33, 37) and the public messaging should instead emphasize that prostate cancer commonly is silent or asymptomatic, particularly in the curable stages of the disease. Men with urinary symptoms should have the same information regarding benefits and harms of screening for prostate cancer."

VERSION 2 – REVIEW

**REVIEWER**	Katy Bell<br>The University of Sydney Faculty of Medicine and Health, School of Public Health
**REVIEW RETURNED**	03-Jun-2024

**GENERAL COMMENTS**	Thank you for your comprehensive and thorough response to my comments, and those of the other reviewers and editors. I only have one very minor suggestion for an edit on page 4 of the manuscript, where you might clarify that family history was defined as a diagnosis in any family member at any age and not just first-degree relatives.

**REVIEWER**	Yiwen Zhang<br>Harvard University T H Chan School of Public Health
**REVIEW RETURNED**	28-May-2024

**GENERAL COMMENTS**	It seems figure legends are missing. Please add titles and descriptions for each of the figures.

**REVIEWER**	Zhengyi Deng<br>Stanford University School of Medicine
**REVIEW RETURNED**	02-Jun-2024

**GENERAL COMMENTS**	In the discussion. The authors mentioned that "Our findings indicate that men overestimate the benefit of screening which is in line with other reports showing that both patients and healthcare workers tend to overestimate the benefits from medical interventions (40, 41)." I didn't see results showing men overestimate the benefits of the screening. Please double check if this statement is supported by your study.

VERSION 2 – AUTHOR RESPONSE

Reviewer: 2
Dr. Yiwen Zhang, Harvard University T H Chan School of Public Health
Comments to the Author:
It seems figure legends are missing. Please add titles and descriptions for each of the figures.

- Reply: Titles are included in the submission system as required and not in the figure files.

Reviewer: 3
Dr. Zhengyi Deng, Stanford University School of Medicine
Comments to the Author:
In the discussion. The authors mentioned that "Our findings indicate that men overestimate the benefit of screening which is in line with other reports showing that both patients and healthcare workers tend to overestimate the benefits from medical interventions (40, 41)." I didn't see results showing men overestimate the benefits of the screening. Please double check if this statement is supported by your study.

- Reply: We have revised the sentence to clarify and is now written as follows *"Our findings show that men overestimate their risk of prostate cancer, and thereby potentially also overestimate the benefit of screening which is in line with other reports showing that both patients and healthcare workers tend to overestimate the benefits from medical interventions (43, 44)"*

Reviewer: 1
Dr. Katy Bell, The University of Sydney Faculty of Medicine and Health
Comments to the Author:
Thank you for your comprehensive and thorough response to my comments, and those of the other reviewers and editors. I only have one very minor suggestion for an edit on page 4 of the manuscript, where you might clarify that family history was defined as a diagnosis in any family member at any age and not just first-degree relatives.

- Reply: We added this clarification as suggested and now reads: *"The survey started with questions about prior PSA testing, prior prostate examinations, and family history of prostate cancer (diagnosis in any family member at any age, not only first-degree relatives) (Supplementary document 1)."*
